# Green-Synthesized Nanomaterials for Water Disinfection: Mechanisms, Efficacy, and Environmental Safety

**DOI:** 10.3390/nano15191507

**Published:** 2025-10-01

**Authors:** Jannatul Ferdush, Md. Mahbubur Rahman, Md Mahadi Hassan Parvez, Md. Abdullah Al Mohotadi, Md. Nizam Uddin

**Affiliations:** 1 Department of Mechanical Engineering, Khulna University of Engineering & Technology, Khulna 9203, Bangladesh; jferdush555@gmail.com (J.F.); mahadihparvez@gmail.com (M.M.H.P.); abdullahalmohotadi@gmail.com (M.A.A.M.); 2 James C. Morriss Division of Engineering, Texas A & M University-Texarkana, 7101 University Ave., Texarkana, TX 75503, USA

**Keywords:** antimicrobial, silver nanoparticle, zinc oxide nanoparticle, cell wall disruption, ROS generation, in vitro and in vivo, cytotoxicity, environmental safety

## Abstract

Safe drinking water is essential, yet millions of people remain exposed to contaminated supplies. Conventional treatments such as chlorination and UV light can kill microbes, but they also create harmful byproducts, face resistance issues, and are not always sustainable. Green-synthesized nanomaterials (GSNMs) are emerging as an eco-friendly alternative. Produced with plants, microbes, algae, and natural polymers, these materials merge nanotechnology with green chemistry. Among them, silver, zinc oxide, copper oxide, titanium dioxide, and graphene-based nanomaterials show strong antimicrobial effects by disrupting membranes, generating reactive oxygen species (ROS), and damaging genetic material. Compared with chemically made nanoparticles, GSNMs are often safer, cheaper, and more environmentally compatible. Nevertheless, concerns about toxicity, environmental fate, and large-scale use remain. This review highlights recent progress in GSNM synthesis, antimicrobial mechanisms, and safety considerations, highlighting their potential to enable sustainable water disinfection while identifying critical areas for further research.

## 1. Introduction

One of the most significant concerns of our day is still access to safe and clean water, particularly as worldwide water supplies continue to be strained by pollution, population increase, and climate change. Access to safe water is a major global concern, increasingly challenging due to pollution, population growth, and climate change. [1]. For example, the 2020 UNESCO development report states that 68% of the world’s population drinks unsafe water, and water contamination has cost the United States $2.2 billion in damages [2].

Although traditional techniques like UV treatment, ozonation, and chlorination guarantee the safety of the water, they are less efficient against resistant infections, use a lot of energy, and produce hazardous byproducts [3]. Again, numerous methods, including solvent extractions, micro- and ultra-filtrations, sedimentation and gravity separations, flotation, precipitation, adsorption, etc., have been studied and applied for water purification over the years [4]. However, the dangerous organic pollutants cannot be entirely eliminated by wastewater treatment facilities; as a result, they are frequently found in waters with concentrations between 15 and 400 ng/L. According to existing studies, these persistent pollutants are detrimental to the ecosystem even at ng/L levels [1]. Researchers are increasingly looking to nanotechnology as a possible solution to get beyond these obstacles. A wide range of dangerous microorganisms can be effectively targeted and neutralized by nanoparticles (NPs) due to their distinct physicochemical characteristics [5]. Among the many strategies, Green-synthesized nanomaterials (GSNMs) are notable for their potent antibacterial activity as well as their biocompatibility, environmental friendliness, and low environmental effect [6]. They are, therefore, becoming a safer and more sustainable way to go forward in the pursuit of universal water security.

**Figure 1:**
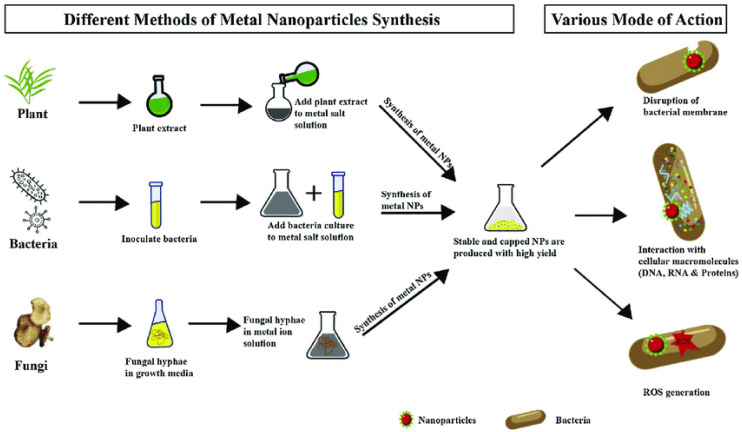
Pictorial representation of green synthesis of metal NPs using plants, bacteria, and fungi, along with their antimicrobial action mechanisms. Reproduced with permission from Singh et al., Biotechnology Reports; published by Elsevier, 2020 [11].

Green synthesis, shown in Figure 1, eliminates the need for dangerous chemicals typically used in physical or chemical synthesis by reducing and stabilizing NPs using biological agents like plant extracts, microbes, or natural polymers [7]. Because nanomaterial surfaces containing bioactive chemicals enhance their antibacterial and environmental compatibility [8]. Silver, zinc oxide, and titanium dioxide NPs produced through environmentally friendly methods are noticeable examples [9]. These NPs have shown strong bactericidal activity against pathogens like *P. aeruginosa*, *S. aureus*, and *E. coli* [10]. A general example of how various biological organisms, including bacteria, fungi, and plants, can produce metal and metal oxide NPs and their antibacterial properties. Their significant antibacterial action against microorganisms, bioactive surface functionality, and environmental compatibility are visibly highlighted [11].

Green-synthesized NPs have antimicrobial properties through a variety of ways [12]. These include direct contact with microbial membranes, reactive oxygen species (ROS) generation to induce oxidative stress, metal ion release, and photocatalytic degradation of microbial structures. Green synthesis strengthens these mechanisms by adding biomolecules that help target and stick to microbial cells [13]. The antimicrobial efficiency of plant-mediated NPs is greatly impacted by their frequent presentation of size homogeneity and shape modulation [14]. These days, a lot of focus is placed on creating various TiO_2_-based nanomaterials for photocatalysis and other uses. It is possible to synthesize nanomaterials in 0D, 1D, 2D, and 3D structures [15]. For instance, the high surface area of spherical TiO_2_ as a 0D-nanomaterial is crucial for both adsorption and photocatalysis. Because of their short distance for charge carrier diffusion, light-scattering capabilities, and ability to create self-standing nonwoven mats, 1D fiber and tube structures can subsequently minimize the possibility of recombination of the photogenerated electron–hole pairs. Additionally, 3D monoliths have high carrier mobility, whereas 2D nanosheets have a smooth surface and strong adhesion.

Compared to their chemically synthesized counterparts, GSNMs have shown more effective disinfection results against Gram-positive and Gram-negative bacteria, viruses, and even protozoa, frequently at much lower doses. For example, under atmospheric settings, green silver nanoparticles (AgNPs) have demonstrated 100% deactivation of *Salmonella* and *E. coli* in a matter of minutes [16]. In a similar way, ZnO and TiO_2_ NPs produced from extracts of *Azadirachta indica* and *Moringa oleifera* have shown outstanding efficacy in breaking down the amount of microbial matter in wastewater without producing harmful residues [17].

Notwithstanding their encouraging performance, before being widely used, the green-synthesized toxicity of nanomaterials and environmental safety profiles need to be thoroughly evaluated. Plant-based capping agents reduce toxicity, but the long-term fate and bioaccumulation of NPs in non-target organisms remain unclear [18]. According to Tan et al. [19], the coating biomolecules can affect the mobility, aggregation, and interaction of NPs with aquatic biota. For risk evaluations to guarantee both disinfection effectiveness and ecological sustainability, life cycle analysis, ecotoxicity profiling, and exposure modeling are necessary [20].

Finally, GSNMs provide reduced toxicity, improved antimicrobial mechanisms, and environmental compatibility, making them a sustainable, inventive, and successful approach to water disinfection. However, to move these nanotechnologies from laboratory experiments to extensive real-world applications, integrated research on mechanism, efficacy, and environmental safety is essential. This study reviews recent advances in green nanomaterials for water disinfection, covering their synthesis, microbial inactivation, effectiveness against pathogens, and environmental impacts.

## 2. Green Synthesis Approaches for Nanomaterials

**Figure 2:**
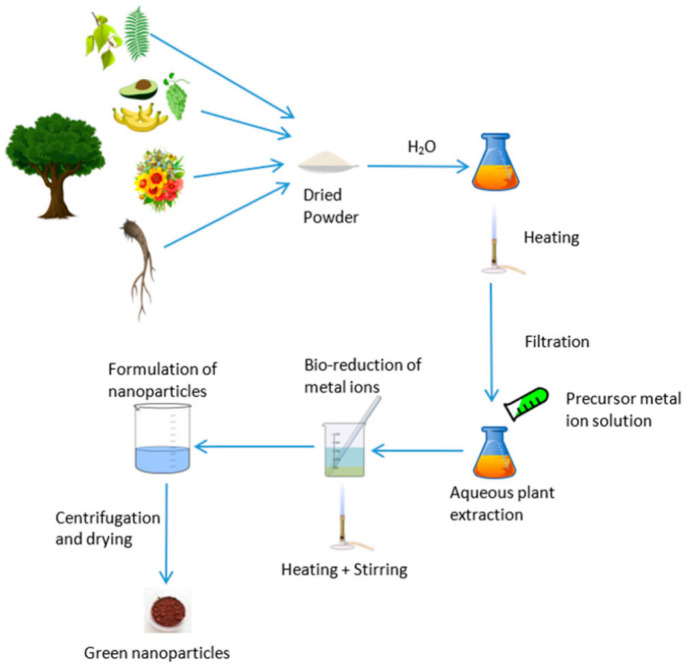
Green synthesis of bio-nanoparticles from plants [23].

The green synthesis of nanomaterials is a sustainable and environmentally friendly substitute for traditional chemical and physical processes, which frequently include hazardous byproducts, excessive energy consumption, and toxic solvents [21,22]. This method uses reducing and stabilizing chemicals that are naturally occurring, mostly from plants, bacteria, fungi, algae, and natural polymers, to create metal and metal oxide NPs in atmospheric circumstances. The process to produce GSNMs, depicted in Figure 2, starts with the preparation of plant extract, then moves on to the creation of NPs by bio-reduction and integration into water purification systems [23]. Unlike chemical methods that rely on harsh or toxic substances, this approach makes use of renewable, biodegradable, and non-toxic resources. That means it is safer for the environment, cost-effective, and easier to work with. At the same time, the nanoparticles produced through the green synthesis approach are more stable, biocompatible, and often show strong antimicrobial activity. This environmentally friendly method is appropriate for decentralized and inexpensive water treatment technologies because it emphasizes the dual benefits of sustainability and antimicrobial effectiveness. In other words, green synthesis not only reduces the environmental footprint but also delivers materials with real practical benefits.

### 2.1. Plant-Mediated Synthesis

Plant-mediated synthesis has become one of the most popular and prospective green synthesis methods for creating metal and metal oxide NPs because of its convenience of use, low cost, beneficial to the environment, and the amount of bioactive phytochemicals that are naturally present. Without the need for dangerous chemical reagents, these phytochemicals, which include polyphenols, terpenoids, flavonoids, saponins, tannins, and alkaloids, act as both stabilizing and reducing agents during the creation of NPs [24]. For this reason, many different types of aromatic and medicinal herbs have been investigated. Because of their strong antimicrobial qualities and diverse phytochemical characteristics, *Azadirachta indica* (neem), *Moringa oleifera*, *Ocimum sanctum* (holy basil), and *Camellia sinensis* (green tea) have all been thoroughly investigated for the synthesis of NPs such as silver (Ag), zinc oxide (ZnO), and titanium dioxide (TiO_2_) [25].

**Figure 3:**
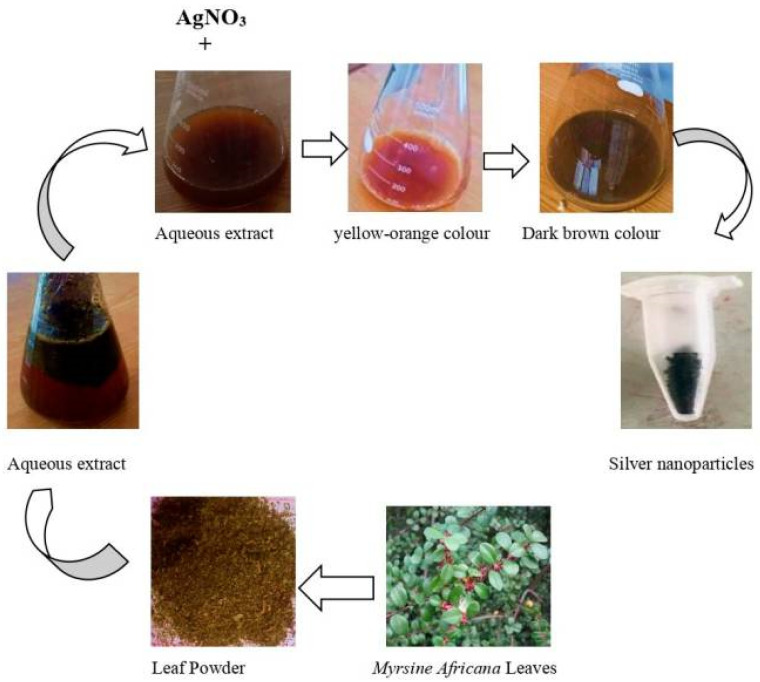
Schematic illustration of silver nanoparticle synthesis employing *Myrsine africana* leaf extract [26].

Phytochemicals found in leaf extracts serve as organic stabilizing and reducing agents during plant-mediated synthesis, promoting the production of nanoparticles. The process of creating silver nanoparticles from *Myrsine africana* leaves is illustrated in Figure 3. The first step in the procedure is gathering and drying the leaves, which are then ground into powder and extracted using water. A noticeable color shift from yellow-orange to dark brown happens when the extract is combined with silver nitrate (AgNO_3_), indicating the reduction of silver ions and the nucleation of nanoparticles [26].

**Figure 4:**
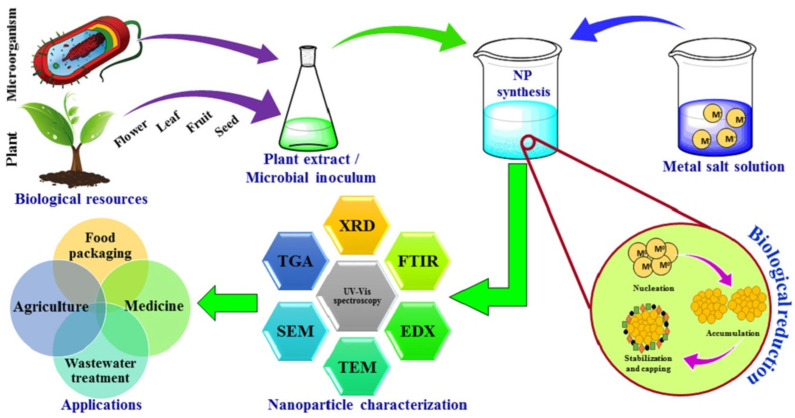
Schematic representation for plant-mediated biosynthesis of NPs [28].

This green synthesis method usually requires combining an aqueous plant extract with a suitable metal salt solution at room temperature or slightly above. Within minutes to a few hours, metal ions undergo bio-reduction, which is frequently signified by a noticeable color shift brought on by surface plasmon resonance (SPR) phenomena. This is especially true for noble metal NPs like silver or gold. For example, a change from pale yellow to dark brown, which correlates to SPR absorption in the 400–450 nm region, clearly confirms the creation of AgNPs [27]. Generally, a metal salt solution at the appropriate pH and temperature is combined with plant biomass or extract to synthesize NPs. The color shift of the solution can be used as the primary confirmation of the NPs’ production. Figure 4 presents the experimental protocol for the synthesis of NPs utilizing plant biomass. In atmospheric settings, metal ions (such as Ag^+^) undergo bio-reduction when combined with plant extracts. For AgNPs, this usually results in a UV–Vis peak in the 400–450 nm region as well as a striking SPR color shift (for example, from pale yellow to brown) [28]. Moreover, Shyam et al. [29] emphasize this color change as a preliminary confirmation of nanoparticle formation. The main benefit of this approach is that its dynamics are significantly greater or comparable to those of chemical nanoparticle manufacturing approaches. The size and stability of the resulting NPs are influenced by variables such as pH, temperature, extract concentration, metal ion concentration, and reaction duration. Therefore, the synthetic process that adheres to all green chemistry principles and may be used for sustainable development can be referred to as this plant-based synthesis [30].

Additionally, according to Ahmed et al. [31], plant-mediated NPs are known to have distinct morphologies and improved biocompatibility when compared to their chemically synthesized competitors. This makes them especially valuable in biomedical applications, antimicrobial coatings, and water disinfection. In addition to lowering toxic effects on the environment, the application of natural plant metabolites adds functional groups to the surface of NPs, which improve their production of reactive oxygen species (ROS) and make it easier for them to connect with microbial membranes [32].

### 2.2. Microbial-Mediated Synthesis (Bacteria and Fungi)

Microorganisms, especially bacteria and fungi, are becoming more and more acknowledged as efficient bio-factories for the synthesis of metal and metal oxide NPs because of their remarkable detoxifying, resistance, and metal ion bioaccumulation capabilities [33]. In contrast to chemical synthesis, microbial-mediated methods use enzymatic reactions and naturally occurring biomolecule releases to produce NPs in a sustainable and environmentally acceptable way [34].

#### 2.2.1. Bacterial-Mediated Nanoparticle Synthesis

Both internal and extracellular synthesis of NPs is possible in bacteria. The biosynthesis of silver (Ag), gold (Au), zinc oxide (ZnO), and selenium (Se) NPs within the cytoplasm or in the culture supernatant has been extensively researched for species like *Pseudomonas aeruginosa*, *Bacillus subtilis*, and *Escherichia coli* [35]. Through their metabolic processes, these microbes mainly use reductase enzymes and biomolecules like proteins, peptides, and NADH [Nicotinamide adenine dinucleotide (NAD) + Hydrogen (H)]-dependent reductases to reduce metal ions. These biomolecules also serve as stabilizing agents [36].

**Figure 5:**
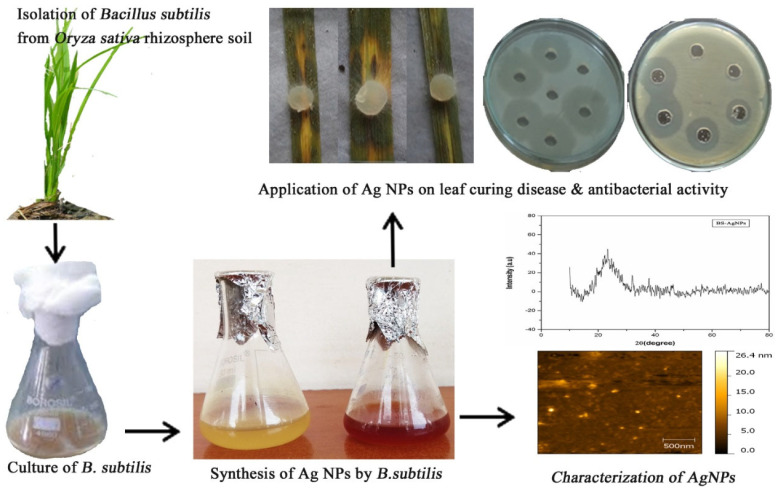
Extracellular biosynthesis of AgNPs by Bacillus species using cell-free supernatant [39].

For example, spherical AgNPs with effective antibacterial properties against aquatic bacteria are produced when *Bacillus subtilis* uses a nitrate reducer to convert Ag^+^ to Ag^0^ [37]. Similarly, ZnO NPs with outstanding photocatalytic and antibacterial qualities have been synthesized using *Escherichia coli* under ambient atmospheric circumstances [38]. Figure 5 depicts the green synthesis process in which silver ions (Ag^+^) undergo reduction to elemental silver (Ag^0^) NPs. This process is verified by a color shift from pale yellow to brown, the appearance of a surface plasmon resonance (SPR) peak in UV–Vis spectra at approximately 450 nm, and morphological characterization using atomic force microscopy (AFM) and scanning electron microscopy (SEM), which reveal spherical NPs that are between 40 and 50 nm in size [39].

#### 2.2.2. Fungal-Mediated Nanoparticle Synthesis

Since fungi can withstand higher metal concentrations, synthesize more extracellular enzymes, and create more biomass, they are frequently preferred over bacteria for the generation of NPs. Fungal systems are particularly ideally suited for efficiently and sustainably increasing the generation of NPs because of these positive effects [40]. The green synthesis of different NPs has been thoroughly studied using fungi like *Aspergillus niger*, *Fusarium oxysporum*, and *Penicillium chrysogenum* [41].

**Figure 6:**
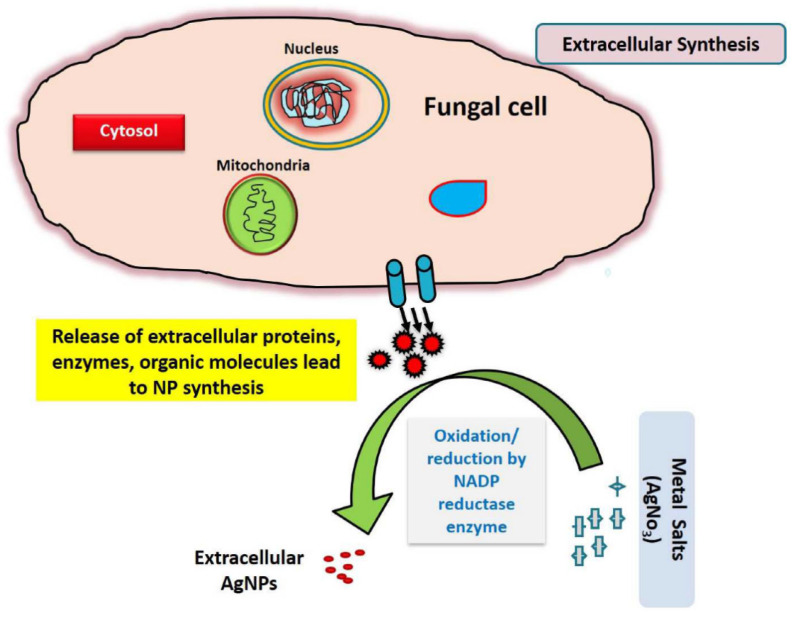
Schematic representation of extracellular fungal-mediated nanoparticle synthesis [45].

According to Iqbal et al. [42], *Aspergillus niger* is specifically capable of bio-synthesizing Ag and Se NPs with excellent stability and potent antibacterial activity against *Candida albicans*, *S. aureus*, and *E. coli*. The concept is that fungal metabolites cap the NPs, improving their distribution and biocompatibility, while coenzymes, such as reductases and proteins, reduce metal ions [43]. Additionally, the extracellular production process is preferred due to its simplicity, ease of recovering NP, and decreased cytotoxicity, where fungal culture filtration reacts directly with metal salt solution [44]. Figure 6 demonstrates how fungi produce NPs through extracellular biosynthesis, where biomolecules like polysaccharides, phenolics, and nitrate reductase that function as both stabilizing and reducing agents. Fungal metabolites, including enzymes and proteins, reduce metal ions (e.g., Ag^+^) to NPs in the extracellular medium, where capping agents stabilize the resulting NPs. When the fungus filtrate interacts with metal salt solutions, color changes that are observable signify the creation of NPs. For large-scale synthesis, the extracellular technique is particularly beneficial since it makes late recovery and purification of NPs easier. To verify synthesis and evaluate the characteristics of NPs, characterization methods such as UV–Vis, DLS, and TEM are frequently employed [45].

### 2.3. Algae-Mediated Synthesis

In the field of green nanotechnology, algae-mediated nanoparticle production has become a popular and effective method. Both clean water and marine algal species, including *Ulva lactuca*, *Sargassum muticum*, *Chlorella vulgaris*, and *Spirulina platensis*, are used in this process because they are abundant in biomolecules that can serve as stabilizing, capping, and reducing agents while generating NPs [46]. Numerous bioactive substances, such as polysaccharides, proteins, amino acids, lipids, vitamins, pigments (such as chlorophylls and phycobiliproteins), and polyphenols, are abundant in these algae and can all contribute significantly to the stabilization of the resultant NPs and the reduction of metal ions [47].

**Figure 7:**
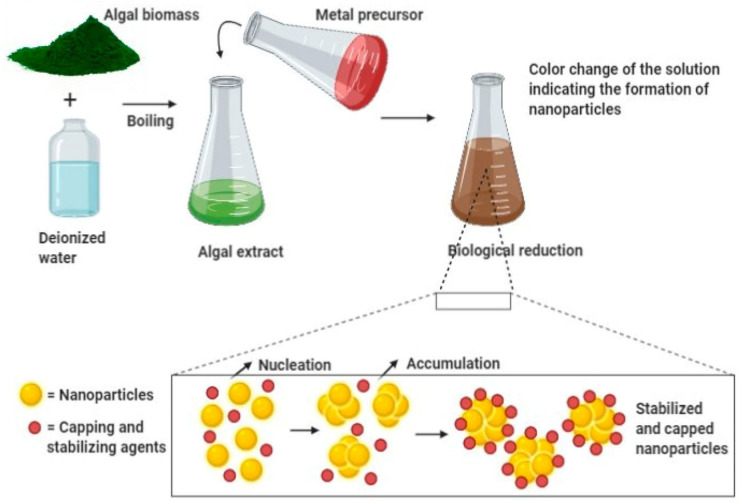
Schematic representation of algae-mediated green synthesis of NPs [48].

Figure 7 illustrates the basic process of creating NPs with extracts from algae. Chlorella vulgaris, Spirulina platensis, and Sargassum muticum are examples of algae that emit bioactive chemicals that stabilize metal ions (such as Ag^+^, Zn^2+^, and Fe^3+^) and reduce them into their corresponding NPs. The method is perfect for use in environmental cleanup and water disinfection because it is energy-efficient, environmentally benign, and consistent with green chemistry principles [48].

In addition to being scalable and affordable, algae-mediated synthesis also helps to regenerate biomass and absorb carbon dioxide, improving its environmental sustainability. The principles of green chemistry are in alignment with this approach, which can be carried out in surroundings and does not call for dangerous chemicals or significant energy components [49].

According to recent studies, NPs made from algae extracts have potent antioxidant, antimicrobial, and catalytic properties, which qualify them for a range of environmental uses. For example, silver NPs made from *Spirulina platensis* demonstrated exceptional effectiveness against *Staphylococcus aureus* and *Escherichia coli*, as well as the degradation of harmful dyes like rhodamine B and methylene blue [50].

Furthermore, it has been tested that algae-mediated nanomaterials are very successful in eliminating heavy metals and waterborne pathogens from contaminated water systems. The adsorption of lead (Pb^2+^), cadmium (Cd^2+^), and arsenic (As^3+^) from industrial wastewater has shown great promise for zinc oxide and iron oxide NPs produced by Chlorella vulgaris [51,52]. Eventually, encouraging their use in sustainable nanoparticle synthesis is the fact that macroalgae, like *Sargassum muticum*, have attracted consideration because of their high metaling capacity and widespread availability in marine environments [53].

### 2.4. Biopolymer and Natural Molecule-Mediated Synthesis

Natural biomolecules such as lignin, gelatin, starch, and chitosan have drawn a lot of interest as green capping and reducing agents in the environmentally friendly synthesis of NPs. These biopolymers are perfect for the manufacture of ecologically friendly and sustainable nanomaterials since they are plentiful, renewable, biodegradable, and non-toxic. They can play a variety of roles during nanoparticle synthesis, including metal ion reduction, particle size control, surface stabilization, and improved antimicrobial activity, owing to their multipurpose qualities, including biocompatibility, film-forming ability, and chelation with metal ions [54].

#### 2.4.1. Chitosan-Mediated Nanoparticles

Chitosan, which is extracted from chitin, exhibits superior antimicrobial properties and colloidal stability in water. According to studies, CuNPs and AgNPs based on chitosan have potent bactericidal properties that are frequently boosted by better dispersion and controlled release [55]. Reviews of Ag and Au NPs coated with chitosan also highlight the particles’ strong antibacterial activity against both Gram-positive and Gram-negative bacteria, as well as their durability and biocompatibility [56].

**Figure 8:**
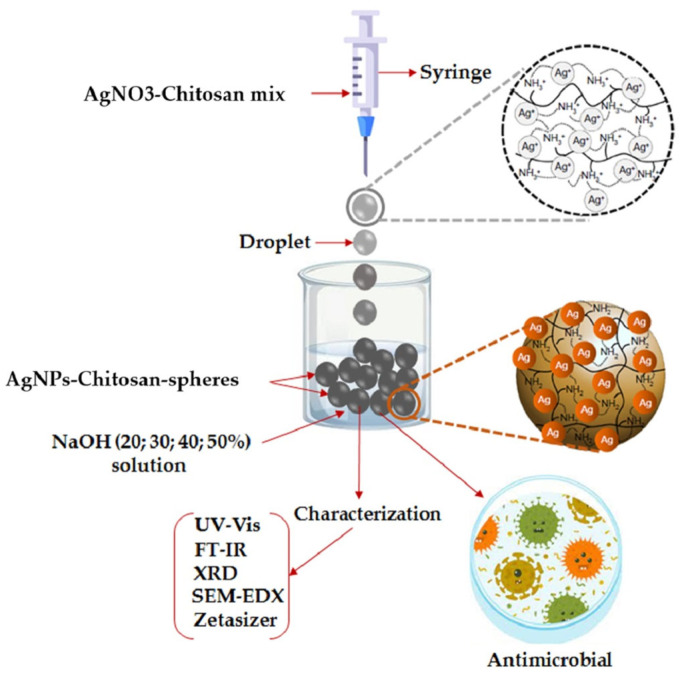
Schematic representation of chitosan–silver nanoparticle (CS/AgNP) synthesis via ionic gelation and chemical reduction [57].

Moreover, these AgNPs and CuNPs mediated by chitosan have demonstrated exceptional antibacterial, antifungal, and antibiofilm properties, rendering them ideal for use in food packaging, water disinfection, and wound healing. Figure 8 represents how chitosan-mediated AgNPs are made in an environmentally friendly manner. Chitosan serves as both a stabilizing and reducing agent, facilitating the formation of AgNPs under mild conditions. The NPs are stabilized through electrostatic interactions and polymeric capping, resulting in enhanced dispersion and antibacterial efficacy. Chitosan’s amine and hydroxyl functional groups stabilize the NPs while interacting with silver ions (Ag^+^) and reducing them to Ag^0^. In order to promote gelation and the creation of NPs, the procedure frequently uses mild alkaline conditions (such as NaOH). The resultant CS/AgNPs are perfect for biomedical and water disinfection applications due to their strong antibacterial qualities, homogeneous particle size, and outstanding colloidal stability [57].

#### 2.4.2. Starch and Gelatin-Mediated Nanoparticles

A cheap, biodegradable polymer made up of amylose and amylopectin, starch has become a promising green capping and reducing agent for metal-based nanoparticle production. Without the need for dangerous chemical reductants, its natural abundance, film-forming capabilities, and abundant hydroxyl functional groups allow for the regulated nucleation and stabilization of NPs in moderate environments. The potential of starch-based AgNPs for use in biomedical and environmental applications has been confirmed by their successful antibacterial properties against *Staphylococcus aureus* and *Escherichia coli* when synthesized at room temperature [58].

**Figure 9:**
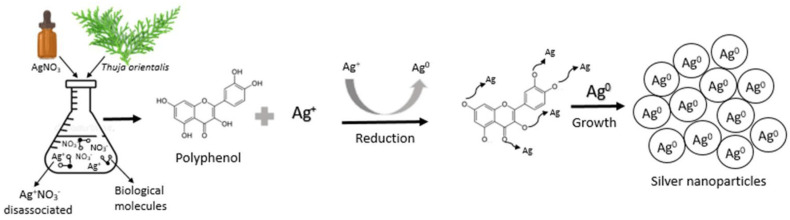
Schematic representation of starch-mediated sol–gel synthesis of metal oxide NPs [60].

Starch-capped AgNPs or ZnO NPs have been effectively incorporated into biodegradable films for use as antimicrobial coatings on food-contact surfaces in food packaging, with significant inhibition of foodborne pathogens like *S. aureus* and *E. coli* [59]. Figure 9 provides a green synthesis route where starch is used in the sol–gel method to fabricate NPs. Starch acts as both a reducing and stabilizing agent, guiding the formation, growth, and dispersion of NPs during the sol–gel process. Starch’s abundant hydroxyl groups provide binding sites for metal ions and facilitate controlled hydrolysis and condensation reactions. During the gelation and drying stages, starch not only promotes the uniform growth of NPs but also functions as a natural capping agent, preventing agglomeration and enhancing colloidal stability [60].

The protein-based biopolymer gelatin, which is produced from collagen, has also been used extensively to create nanocomposite biofilms that include silver, gold, or zinc NPs. It can be used alone or in conjunction with chitosan. These composites can be used for wound dressing and active food packaging because they have improved mechanical strength, flexibility, and controlled nanoparticle release [61]. Gelatin’s amino and carboxyl groups aid in metal ion chelation and reduction, which enhances the stability of NPs and their antimicrobial activity.

#### 2.4.3. Lignin and Other Natural Molecules

**Figure 10:**
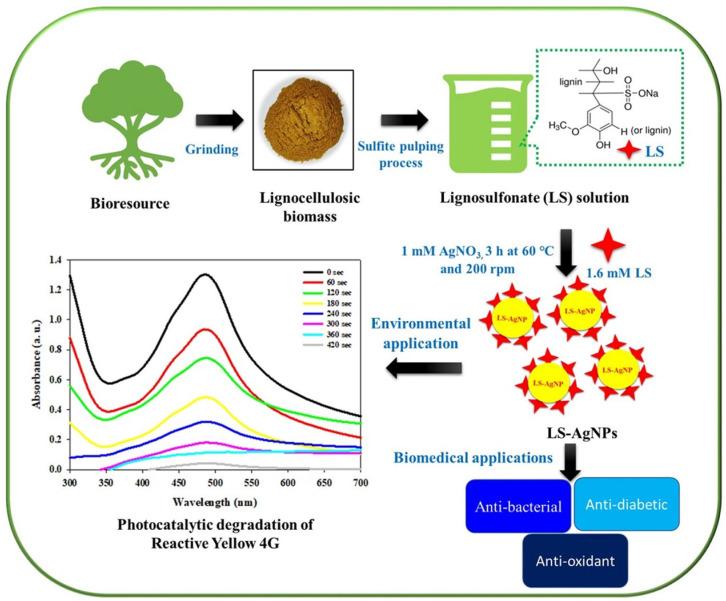
Schematic illustration of AgNPs synthesis using sodium lignosulfonate (a lignin derivative) as a green reducing and stabilizing agent [65].

Although lignin and other plant-derived polyphenolic macromolecules are not as commonly employed as chitosan or starch, they are becoming important instruments in the environmentally friendly creation of NPs. Rich in functional groups including phenolic hydroxyl, carboxyl, and methoxy groups, lignin is a complex aromatic polymer that is widely found in plant cell walls. Because of these chemical characteristics, lignin can function as a stabilizing agent, which helps regulate particle size and inhibit aggregation, in addition to being a natural reducing agent, which turns metal ions into NPs. Because it is polyphenolic, electrons can more easily move to metal ions, forming stable NPs with desired characteristics [62]. According to Iravani et al. [63], lignin derived from agricultural waste has been effectively utilized to create a range of NPs, such as AgNPs, magnetite (FeO_4_), and zinc oxide (ZnO). These lignin-based nanomaterials have shown encouraging antimicrobial and antioxidant properties, indicating their potential applications in environmental remediation, biosensing, and water purification. Beyond its practical uses, lignin-mediated synthesis provides a means of converting biomass waste into valuable nanomaterials, supporting the concepts of waste valorization and the circular bioeconomy [64]. Figure 10 illustrates the environmentally friendly synthesis of AgNPs using sodium lignosulfonate, which, because of its abundance of phenolic and carboxyl functional groups, functions as a natural reducing agent and stabilizer. The reduction of Ag ions to AgNPs is the first step in the process, which is then stabilized by the functional groups in lignin. By facilitating electron transport to silver ions, the biopolymer makes it possible to manufacture environmentally acceptable NPs without the need of dangerous chemicals. The resultant AgNPs are appropriate for antimicrobial uses, such as water disinfection, due to their improved stability and consistent size distribution [65].

All things considered, the green synthesis of nanomaterials mediated by biopolymers provides a scalable, affordable, and sustainable substitute for traditional chemical synthesis techniques. These naturally occurring compounds improve the functional performance of NPs for use in water purification, antimicrobial coatings, biosensors, and catalysis while removing the requirement for hazardous predecessors and lessening the environmental impact.

Despite the fact that many studies demonstrate the environmentally beneficial properties of GSNMs, there is frequently a lack of systematic and quantitative assessment. Sustainability can be measured using the 12 principles of green chemistry, but particular indicators are needed for practical comparison [66]. The objective evaluation of greenness across synthesis techniques is made possible by metrics including atom economy (AE), environmental factor (E-Factor), process mass intensity (PMI), energy usage, and toxicity of reagents/byproducts [67]. The contrasting findings of GSNM synthesis methods demonstrate how their sustainability profiles fit nicely with green chemistry concepts. Although biomass leftovers raise the overall E-factor, plant-mediated synthesis provides an excellent compromise between scalability and environmental friendliness [68]. Although microbial-mediated methods using fungi or bacteria are biocompatible, they require prolonged cultivation and nutrient-rich media, which raises PMI and energy consumption [69]. Algae-mediated synthesis, on the other hand, is the most environmentally friendly since it uses renewable feedstocks, produces non-toxic metabolites, and produces little waste, yet harvesting biomass on a large scale is still difficult [70]. However, polymer residues and the usage of chemical crosslinkers may lessen the environmental benefit of biopolymer and natural molecule-mediated techniques, which offer stable nanoparticle creation and sustainable pathways [71]. Collectively, these studies show how criteria like toxicity, PMI, E-factor, and atom economy offer an impartial perspective for assessing and reflecting the general greenness of GSNM synthesis methods. Table 1 summarizes the comparative greenness of major GSNM synthesis approaches.

## 3. Mechanisms of Antimicrobial Action

GSNMs degrade microbial integrity and prevent their survival in water systems through a range of physical, chemical, and biological processes, exhibiting strong antimicrobial activity. These processes mostly rely on the physicochemical characteristics of nanomaterials, which are heavily impacted by the green synthesis approach. These characteristics include size, shape, surface charge, and composition.

### 3.1. Disruption of Cell Membrane Integrity

The disruption of microbial cell membrane integrity is one of the most common and important ways that green-synthesized NPs exhibit antimicrobial action. An essential barrier that controls the entry and exit of ions, nutrients, and waste materials is the cell membrane. When the physiological functions of microorganisms are disrupted, it frequently results in cell death [76].

Green-synthesized metal and metal oxide NPs, including TiO_2_, CuO, ZnO, and Ag exhibit strong antimicrobial activity by electrostatically binding to the negatively charged bacterial surface, initiating membrane destabilization and pore formation [77]. While NPs frequently have a positive surface charge or polar functional groups added during green synthesis, which increase their attraction for microbial surfaces, bacterial membranes normally carry a net negative charge [78].

After adhering, these NPs cause membrane destabilization, hole development, and the leakage of intracellular materials like proteins, ions, and nucleic acids by directly disrupting the lipid bilayer. Membrane disruption is often exacerbated by oxidative stress, further accelerating cell lysis [79].

For instance, transmission electron microscopy (TEM) studies confirmed that AgNPs made with *Azadirachta indica* leaf extract exhibited increased interaction with the membranes of *Escherichia coli* and *Staphylococcus aureus*, leading to visible morphological damage, such as membrane rupture and corrugation [80]. Similar results were seen with ZnO NPs made with Moringa oleifera, which demonstrated ion leakage and selective membrane targeting, demonstrating their potent antimicrobial properties [81]. Research by Mobarak et al. [82] demonstrates that CuO NPs biosynthesized using fungal or algae extracts can cause surface disintegration and membrane pitting in both Gram-positive and Gram-negative bacteria, indicating a broad-spectrum mode of action.

**Figure 11:**
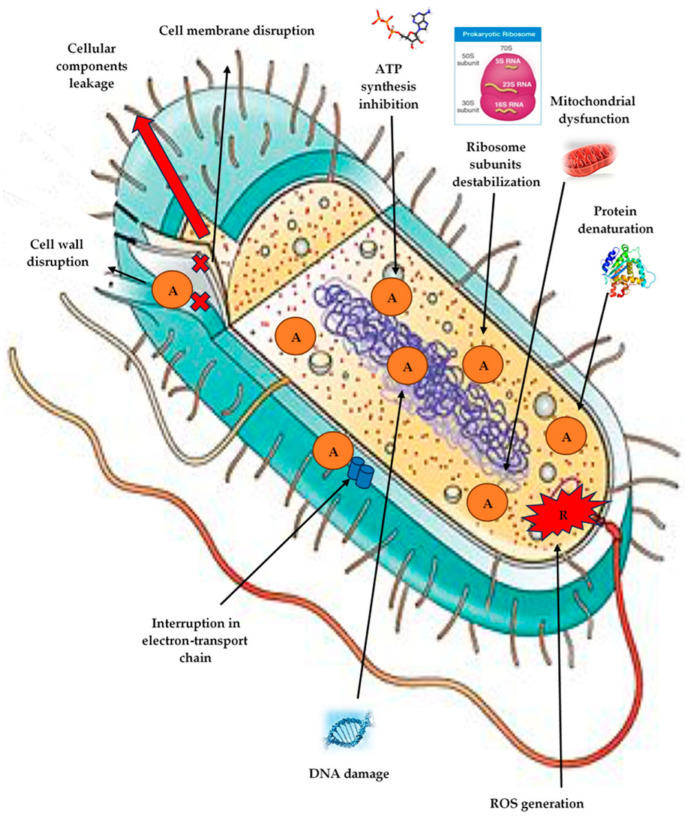
Schematic representation of nanoparticle-induced bacterial cell membrane disruption [84].

Furthermore, SEM imaging and fluorescence-based dye leakage tests indicate that green-synthesized NPs can raise the permeability of membranes, which can result in cytoplasmic leakage and unregulated ion exchange. For example, higher propidium iodide uptake in bacterial cells due to biosynthesized TiO_2_ NPs indicates substantial membrane compromise [83]. The basic antimicrobial mechanism of green-synthesized metal NPs, like ZnO NPs and AgNPs, is demonstrated in Figure 11 with an emphasis on direct interaction with the bacterial cell membrane [84]. In green-synthesized NPs, phytochemicals and natural polymers on the surface provide functional groups that enhance electrostatic attraction to the negatively charged bacterial membrane, allowing the particles to anchor more effectively. Once attached, the metallic or metal oxide core (such as Ag, ZnO, or TiO_2_) perturbs the lipid bilayer, leading to pore formation, leakage of intracellular contents, and eventual cell lysis. As illustrated in the schematic, the capping chemistry promotes close contact with the microbial surface, while the nanoparticle core delivers the mechanical and chemical stress that disrupts membrane integrity.

Cell lysis and bacterial inactivation result from this, along with the loss of vital cytoplasmic contents and cellular potential. Their effectiveness in water disinfection applications is largely dependent on this mechanism [85].

These findings imply that green-synthesized NPs are effective and selective at targeting microbial membranes without endangering mammalian cells, which makes them promising agents for biomedical and water disinfection uses. Plant or microbial extracts’ surface chemistry, size, shape, and capping agents are important factors in regulating the degree of membrane disruption and ought to be tailored for particular uses [86].

### 3.2. Generation of Reactive Oxygen Species (ROS)

The generation of reactive oxygen species (ROS), which include extremely reactive molecules like hydroxyl radicals (•OH), superoxide anions (O_2_•^−^), hydrogen peroxide (H_2_O_2_), and singlet oxygen (^1^O_2_), is another important antimicrobial mechanism of green-synthesized NPs [87]. Major biomolecules like lipids (by peroxidation), proteins via oxidation or denaturation, and nucleic acids via strand breaks or base changes are irreversibly damaged by the oxidative stress that these ROS cause to microbial organisms [88]. It has been extensively demonstrated that green-synthesized ZnO, TiO_2_, Ag, CuO, and FeO_4_ NPs produce ROS using either inherent redox activity or photocatalytic processes. Semiconducting NPs like ZnO and TiO_2_ can enhance ROS production via photocatalysis under UV or visible light, which can be further improved using plant-based biosynthesis, such as *Aloe vera* or *Moringa oleifera* extracts [89,90].

**Figure 12:**
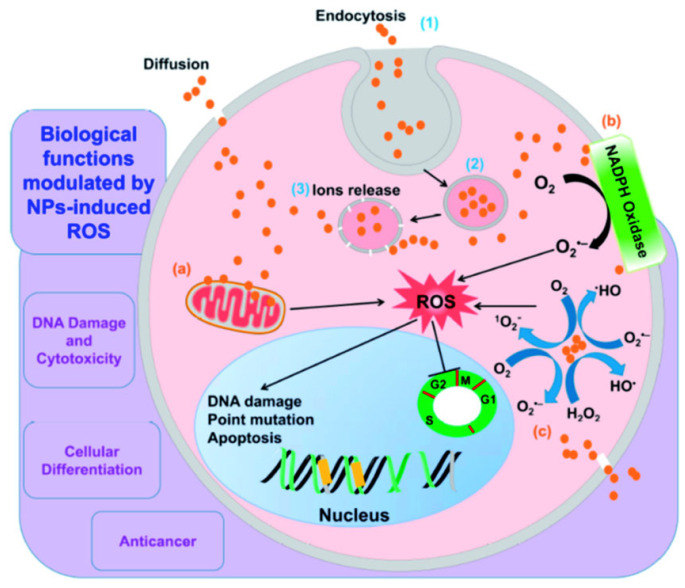
Mechanism of ROS generation by green-synthesized NPs NPs can be internalized into the cell by (1) endocytosis; (2) formation of the endocytotic vesicles; and (3) release of particle ions from vesicles into the cell. The main factors responsible for ROS generation by NPs include: (**a**) interaction with the mitochodria; (**b**) interaction with NADPH oxidase; and (**c**) factors related to the physicochemical properties (size, shape, photoreactive properties, and surface chemistry). These factors lead to ROS generation and its consequences, including DNA damage, cell cycle arrest, alterations in apoptosis, and damage to the cell membrane [91].

Figure 12 clearly focuses on demonstrating how green-synthesized metal and metal oxide NPs generate reactive oxygen species (ROS) and how they contribute to disinfection and bacterial cell damage. It describes the main processes that produce ROS and are fueled by metallic NPs, specifically ZnO NPs and AgNPs, which are produced using environmentally friendly methods [91]. These ROS break through bacterial membranes, oxidize intracellular materials, and eventually cause microorganisms to undergo programmed cell death or apoptosis-like processes [92]. Green capping agents supply functional groups that stabilize nanoparticles and influence electron transfer, thereby complementing the nanoparticle core’s ability to generate ROS under physiological or light-exposed conditions. Furthermore, by oxidizing thiol groups in enzymes and upsetting electron transport chains, green-synthesized AgNPs made from polyphenol-rich plant extracts such as *Azadirachta indica* and *Camellia sinensis* can catalyze Fenton-like reactions and produce H_2_O_2_, which disturbs bacterial physiology [93].

Oxidative stress has been identified as a prominent route in studies that use ROS scavengers such as mannitol, catalase, and N-acetyl cysteine (NAC). The antibacterial effectiveness of the NPs dramatically decreased when ROS were neutralized, suggesting that oxidative damage caused by ROS is a major mechanism of action [94].

Additionally, when biosynthesized using microbial or algal systems, Fe_3_O_4_ and CuO NPs have demonstrated increased Fenton-like reactivity, which enhances bactericidal activity in opposition to multidrug-resistant (MDR) strains and contributes to the in situ generation of hydroxyl radicals [95].

#### 3.2.1. ROS Generation Pathway

Green-synthesized NPs exert antimicrobial activity largely through the generation of reactive oxygen species (ROS) such as hydroxyl radicals (•OH), superoxide anions (O_2_•^−^), and hydrogen peroxide (H_2_O_2_).

#### 3.2.2. Photocatalytic ROS Generation (Semiconductors like ZnO and TiO_2_)

Under light irradiation, photocatalytic ROS production is a prominent mechanism for semiconducting NPs such as ZnO and TiO_2_ [96]. These NPs produce electron–hole pairs (e^−^/h^+^) when they absorb photons (hv). The excited electron then travels to the conduction band, leaving a hole in the valence band. These charge carriers take part in redox reactions: the electron reduces molecular oxygen to superoxide anion (e^−^ + O_2_ → O_2_•^−^), which can then change into H_2_O_2_ and break down into more hydroxyl radicals, while the hole oxidizes water molecules to produce •OH (h^+^ + H_2_O → •OH). Oxidative stress and microbial cell damage are caused by this chain reaction of ROS formation [97].

Light absorption (for semiconductors like ZnO, TiO_2_):

ZnO/TiO_2_ + hν (light)→ e^−^ + h^+^

h^+^ + H_2_O → •OH

e^−^ + O_2_ → O_2_•^−^ → H_2_O_2_ → •OH

**Figure 13:**
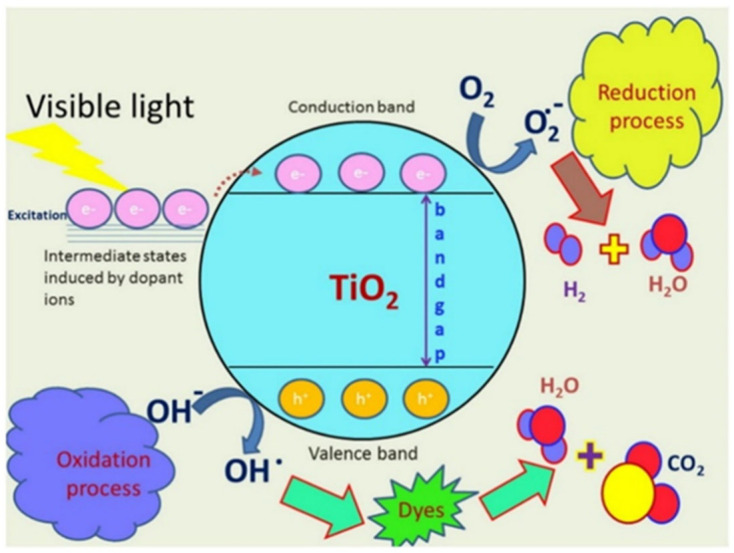
Mechanism of photocatalytic ROS generation by TiO_2_ under light irradiation (hν) [98].

Figure 13 portrays the photocatalytic ROS generation mechanism by metal–ion modified TiO_2_ NPs and its antimicrobial effect via membrane disruption. Upon light irradiation (hν), these semiconducting NPs generate electron–hole pairs (e^−^/h^+^). The excited electrons reduce oxygen (O_2_) to form superoxide (O_2_•^−^), while the holes oxidize water to generate hydroxyl radicals (•OH). These ROS attack bacterial membranes, disrupting lipid bilayers, increasing permeability, and ultimately leading to microbial cell death [98].

Green-synthesized ZnO and TiO_2_ nanoparticles were assessed under UV-A or simulated solar irradiation. Light intensities of 10–50 mW/cm^2^ in the UV-A region (315–400 nm) or solar simulators with 200–800 nm spectra are commonly used to define conditions. For example, Aloe vera–ZnO NPs exhibited more ROS production upon UV-A light irradiation at 365 nm [99], and *Chlorella vulgaris,* which is TiO_2_ NPs, exhibited excellent antibacterial activity upon natural sunlight exposure (spectrum 200–800 nm) [100]. All these facts indicate that narrow-band UV, as well as broad-spectrum solar light, have been utilized in the assessment of photocatalytic activity.

The most important photocatalytic disinfection experiments with green-synthesized TiO_2_ and ZnO nanoparticles comprised proper control experiments. Uncapped TiO_2_ and uncapped ZnO samples prepared in the absence of green capping agents were examined under similar irradiation conditions as for the green-synthesized samples. All controls evidenced systematically reduced antibacterial and photocatalytic activity compared to their corresponding green-synthesized counterparts, validating that the higher performance results from bioactive capping and surface functionalization.

#### 3.2.3. Fenton-like ROS GENERATION (Metal-Based Nanoparticles like Fe_3_O_4_, CuO, AgNPs)

Meanwhile, metal-based NPs, including AgNPs, CuO, and FeO_4,_ are a major generator of ROS due to Fenton-type reactions [101]. These NPs catalyze reactions with H_2_O_2_ in aqueous settings, producing •OH. For example, Cu^+^ ions engage in a Fenton-like process (Cu^+^ + H_2_O_2_ → Cu^2+^ + •OH + OH^−^), whereas Fe^2+^ ions go through a conventional Fenton reaction (Fe^2+^ + H_2_O_2_ → Fe^3+^ + •OH + OH^−^). Also, Ag can enhance ROS formation, especially in the presence of polyphenolic stabilizers. The breakdown of H_2_O_2_ can also be improved by AgNPs stabilized with plant polyphenols, which indirectly generate ROS [102].

These pathways are beneficial for water disinfection applications where light is in short supply because they work especially well in dark environments. Both processes eventually cause microbial membranes, proteins, and nucleic acids to degrade oxidatively, which kills the cell [103].

Fenton-like activity (for Fe_3_O_4_, CuO, AgNPs):

Cu^+^ + H_2_O_2_ → Cu^2+^ + •OH + OH^−^

Fe^2+^ + H_2_O_2_ → Fe^3+^ + •OH + OH^−^

Ag^+^ or Ag^0^ + H_2_O_2_ → Ag^+^ + •OH (indirect)

**Figure 14:**
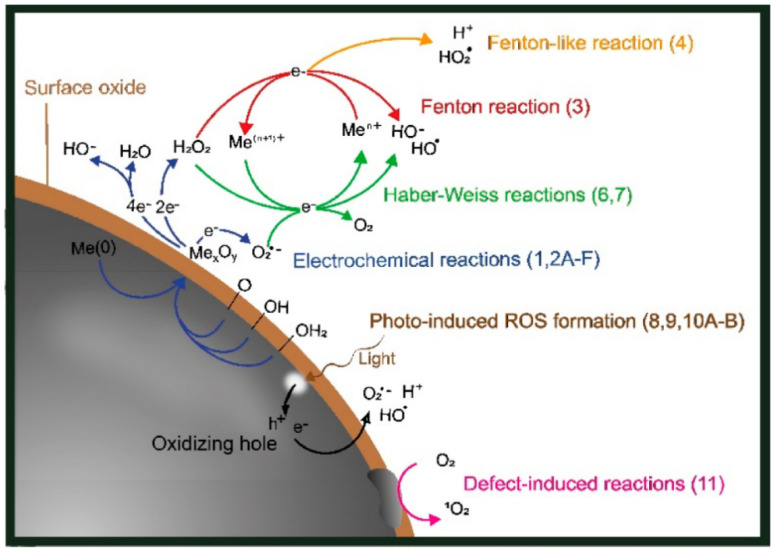
Diagrammatic representation of the several processes that produce ROS in solution and on the surface of oxidized metal particles or large surfaces [104].

Figure 14 displays a schematic representation of the several ROS-generating pathways for metal particles with surface oxides (core-shell particles). The Fenton and Fenton-like ROS generation mechanisms generated by metal and metal oxide NPs (such as FeO_4_, CuO, and AgNPs) are shown. These processes produce highly reactive hydroxyl radicals (•OH), a crucial part of oxidative disinfection, when metal ions combine with hydrogen peroxide (H_2_O_2_) [104].

These ROS cause oxidative stress, membrane integrity loss, and ultimately microbial inactivation by attacking the proteins, DNA, and cell membranes of bacteria. The antibacterial activity of metal-based NPs that are green-synthesized in water disinfection systems is largely dependent on this process [105]. Table 2 summarizes the key biological effects of reactive oxygen species (ROS) generated by green-synthesized NPs. It highlights how different ROS types contribute to the disruption of essential cellular components, ultimately leading to microbial cell death.

### 3.3. Metal Ion Release

Another crucial antimicrobial action of GSNMs is their ability to function as reservoirs for bioactive metal ions like zinc (Zn^2+^), copper (Cu^2+^), and silver (Ag^+^), which slowly permeate into the environment [110]. Ag^+^ ions released from biosynthesized nanoparticles disrupt microbial metabolism by interacting with thiol (-SH) groups in vital enzymes and proteins, inhibiting the respiratory chain, denaturing proteins, binding to DNA to prevent transcription and replication, and inducing lipid peroxidation and protein oxidation, ultimately causing microbial cell death in both Gram-positive and Gram-negative bacteria [111,112]. In a similar vein, Cu^2+^ ions can produce ROS in situ through Fenton-like processes and interfere with proton gradients to alter membrane potential, further impairing the production of cellular energy [113].

The surface chemistry, crystallinity, and capping agents obtained during green synthesis have a significant impact on the rate and magnitude of metal ion release. Plant-based reducing agents that contain polyphenolic chemicals, for instance, can stabilize NPs while permitting regulated ion dissolution [114]. Green-synthesized NPs are particularly well-suited for environmental and biomedical applications because of their gradual and continuous ion release, which guarantees extended antimicrobial efficacy while reducing toxicity to non-target organisms [115].

In addition to quantifying ion leaching profiles, studies employing ion-selective electrodes, atomic absorption spectroscopy (AAS), and ICP-MS have shown that adjusting green synthesis factors like pH, plant extract concentration, and temperature during calcination can result in variable release profiles [116]. Moreover, fluorescence microscopy and ion mapping techniques have demonstrated that Ag^+^ and Zn^2+^ ions accumulate in microbial membranes and cytoplasm, leading to membrane depolarization and internal oxidative stress [117].

**Figure 15:**
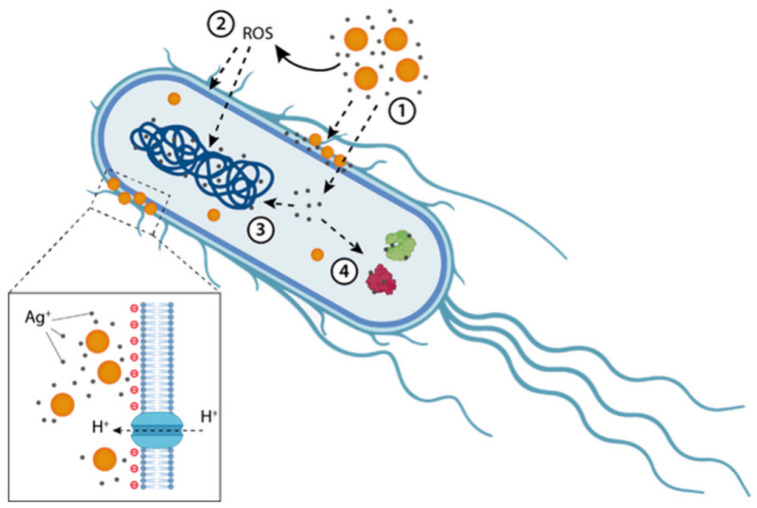
Schematic of silver nanoparticle (AgNP) action via controlled Ag^+^ ion release [120].

The overall function of metal ion release occurs in two ways: it increases indirect oxidative stress by producing ROS and provides direct antimicrobial activity through metabolic interference. Optimizing this mechanism using environmentally friendly synthesis techniques reduces negative environmental effects while increasing antimicrobial potency [118]. Figure 15 depicts how AgNPs release silver ions that interact with the microbial cell, leading to enzyme inhibition, DNA binding, lipid peroxidation, and indirect ROS generation, ultimately resulting in membrane damage and cell death [106]: (1) Acidic and aerobic environments encourage the release of silver ions. (2) The generation of ROS, which subsequently harms DNA and membrane lipids. (3–4) Membrane damage may favor the uptake of Ag^+^ (though they may also enter through membrane channels). Biological capping layers regulate the dissolution of ions from the nanoparticle core; this controlled ion release, together with stabilizing biomolecules, enhances antimicrobial potency while reducing uncontrolled toxicity. Ag^+^ ions have the ability to bind intracellular proteins and the bacterial chromosome after they enter the cytoplasm, which can affect replication and metabolic activities. Higher local dosages of NPs may result from positively charged AgNPs being drawn to negatively charged bacterial membranes. This is where the proton motive force occurs, which lowers the pH locally. This may encourage AgNPs to dissolve even more, raising the concentration of Ag^+^ locally. A Gram-negative bacterium has been used as the model microorganism in this image [119].

When it comes to controlling the antibacterial activity and environmental safety of GSNMs, capping agents are essential. They shape disinfection efficiency and long-term aquatic stability by modifying the surface chemistry of nanoparticles, which in turn controls the production of reactive oxygen species (ROS) and the release of antimicrobial metal ions. Using sample quantitative examples from recent studies, Table 3 summarizes the effects of various classes of natural capping agents, such as proteins, polyphenols, lipids or terpenoids, and polysaccharides, on ROS generation, ion release, and overall antimicrobial results.

Overall, the comparison shows that capping agents have two sides: some (like polyphenols and chitosan-based polysaccharides) can increase antimicrobial potency by slowing ion release or suppressing ROS, while others (like proteins and lipids) prefer stability and biocompatibility over acute toxicity. Optimizing green nanomaterials that strike a balance between high water disinfection efficacy and lower ecological concerns in long-term applications requires a sophisticated understanding of these relationships.

### 3.4. Intracellular Penetration and DNA Damage

Intracellular penetration is one of the more sophisticated antimicrobial techniques of green-synthesized NPs, especially by ultrasmall particles (<20 nm). These NPs can readily penetrate through microbial cell walls and membranes, avoiding external defense barriers, because of their nanosize and surface functionalization, which is obtained from biogenic capping agents [124]. NPs can interact with intracellular targets after they are inside the cytoplasm, which can result in microbial death and serious cellular malfunction [125].

NPs can interact with ribosomes, connect straight up to DNA or related proteins, and accumulate close to the nucleoid region after breaking through the cell membrane. These interactions can lead to genotoxic consequences, including DNA fragmentation, chromosomal condensation, and base oxidation, as well as ribosomal inactivation and protein synthesis suppression [126]. For example, gel electrophoresis and DAPI staining have confirmed that green-synthesized AgNPs utilizing extracts from Azadirachta indica and Camellia sinensis can localize inside bacterial cells and cause DNA cleavage and condensation in *E. coli* and *S. aureus* [127].

**Figure 16:**
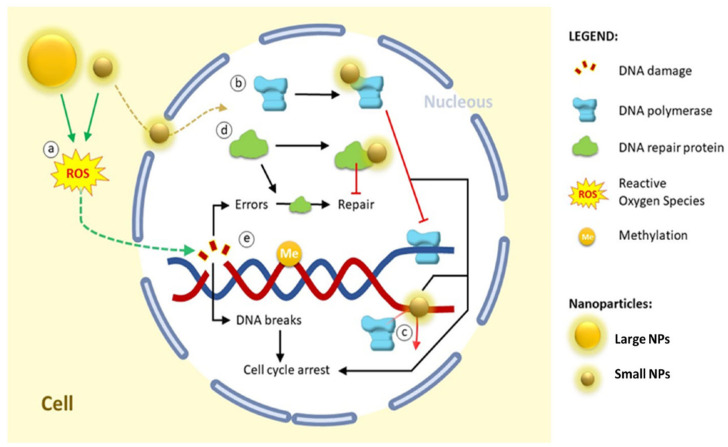
Size-dependent internalization of NPs and consequent impacts on DNA and cellular machinery (**a**) Large NPs cannot be internalized in the nucleus, although they can generate ROS and indirectly induce DNA damage. Small NPs can also generate ROS and can additionally be internalized and interact with nuclear proteins. (**b**) Interaction with DNA polymerase stops replication by inhibiting the interaction of DNA polymerase and DNA; and additionally, (**c**) NPs can bind to DNA and inhibit replication by blocking the binding of DNA polymerase and DNA. In cases where endogenous or exogenous DNA damage (NPs effect) is generated, this can be repaired by DNA damage repair proteins; however, (**d**) some NPs are able to interact with these repair proteins, blocking the repair process. These alterations lead to DNA damage accumulation and, as a consequence, cell cycle arrest; (**e**) additionally, several NPs can also modify the methylation profile (epigenetic alterations) [131].

According to Suganthy et al. [128], AuNPs produced by environmentally friendly methods, including Terminalia arjuna bark extract, have also been demonstrated to enter cells and interact with bacterial genomic DNA, resulting in double-strand breaks and disrupting transcription and replication machinery. Visual proof of NPs within bacterial cells has been obtained by TEM and confocal imaging studies. These particles are frequently observed in close proximity to DNA regions or places that are rich with ribosomes [129]. Crucially, the green synthesis strategy contributes to improving intracellular absorption. Plant metabolite-derived surface biofunctional groups, like flavonoids or tannins, imitate natural ligands to enhance biocompatibility and promote passive or active internalization [130]. Green-synthesized NPs are, therefore, attractive agents against multidrug-resistant infections because of their ability to penetrate cells and cause DNA and ribosome damage, which constitutes a multi-targeted antimicrobial pathway. This process adds to the overall bactericidal efficiency by enhancing the pathways of oxidative stress and membrane damage. Figure 16 highlights the impact of NPs on DNA. In general, internalization in the nucleus depends on size: (a) Despite their ability to produce ROS and indirectly cause DNA damage, large NPs cannot be internalized in the nucleus. In addition to being internalized and interacting with nuclear proteins, small NPs can also produce ROS. (b) Interaction with DNA polymerase prevents replication by preventing DNA polymerase and DNA from interacting; furthermore, (c) NPs have the ability to attach to DNA and prevent replication by preventing DNA polymerase and DNA from binding. DNA damage repair proteins can repair endogenous or exogenous DNA damage (NPs effect); nevertheless, (d) some NPs can interact with these repair proteins and prevent the repair process. These changes result in the buildup of DNA damage and, consequently, cell cycle arrest; (e) a number of NPs can also alter the methylation profile (epigenetic alterations) [131].

### 3.5. Biofilm Inhibition and Quorum-Sensing Disruption

The structured microbial communities known as biofilms have been embedded in a matrix of extracellular polymeric substance (*EPS*), which is made up of proteins, DNA, and polysaccharides. Because of this matrix’s ability to operate as a physical and chemical barrier, bacteria in biofilms are up to 1000 times more resistant to common antibiotics and disinfectants [132]. Nonetheless, GSNMs have shown strong potential in interfering with bacterial communication systems and biofilm formation, particularly those that are small, surface charge modulated, and have bioactive coatings derived from plants [133].

**Figure 17:**
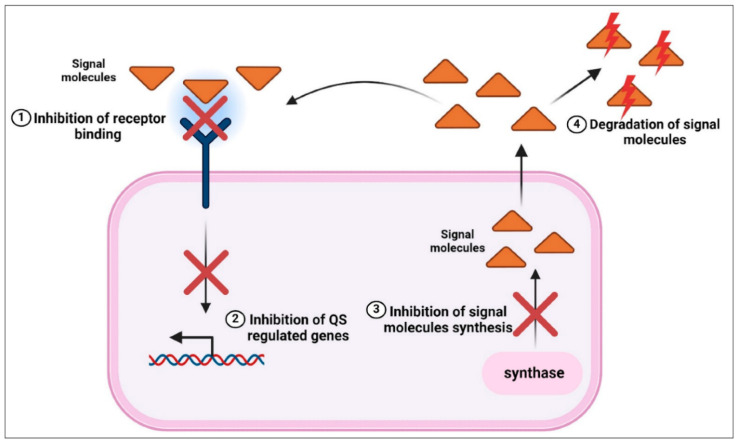
A diagram showing different methods in which NPs could interfere with the QS process [136].

The bacterial cell-to-cell communication system known as quorum sensing (QS), which is in charge of biofilm formation, virulence control, and population coordination, can be disrupted by these NPs, which can also break through the biofilm matrix and interfere with EPS production [134]. Signaling molecules, including autoinducing peptides (AIPs) in Gram-positive bacteria and acyl-homoserine lactones (AHLs) in Gram-negative bacteria, are involved in quorum sensing. NPs can reduce bacterial colonization and pathogenicity by inhibiting biofilm development, maturation, and dissemination through binding to or destroying these signaling molecules [135]. The mechanisms by which green-synthesized NPs prevent bacterial biofilms and interfere with QS systems are presented in Figure 17. In both Gram-positive and Gram-negative bacteria, autoinducers (AHLs or peptide signals) are adsorbed or degraded by NPs, preventing the transmission of QS signals. This leads to the suppression of QS-regulated gene expression, including virulence factors, EPS synthesis, and the creation of biofilm matrix. Reduced bacterial aggregation, poor biofilm development, and increased bacterial vulnerability to environmental stressors and antimicrobial drugs are the results of inhibiting QS activation [136].

Plant-based silver NPs (AgNPs) made from leaf extracts of Eucalyptus globulus and Azadirachta indica [137], for instance, have demonstrated potent antibiofilm activity against pathogens such as Pseudomonas aeruginosa, Escherichia coli, and Staphylococcus aureus [138]. Furthermore, polymer-stabilized NPs such as AgNPs coated with chitosan or starch improve penetration depth and retention, enhance interaction with the negatively charged biofilm matrix, and increase their efficacy in breaking up established biofilms. These NPs have been demonstrated to cause the detachment of sessile bacterial cells, decrease biomass as determined by crystal violet assays, and disrupt the architecture of biofilms [139]. Also, it has been discovered by Vetrivel et al. [140] that green-synthesized ZnO NPs and CuO NPs significantly reduce the ability of P. aeruginosa to form biofilms by downregulating quorum-sensing-regulated virulence factors like pyocyanin, elastase, and rhamnolipid. Green-synthesized NPs’ anti-quorum-sensing capabilities have also been shown in living environments such as zebrafish and plant root colonization tests, as well as co-culture models [141].

In the research of Roy et al. [142], the bioorganic capping agents found on green-synthesized NPs, such as polyphenols, terpenoids, and flavonoids, frequently have anti-QS and anti-biofilm qualities of their own. These properties work in concert with the metallic core to increase efficacy while preserving biocompatibility.

Therefore, by concentrating on both the physical biofilm structures and the underlying indicating networks that regulate their development, green-synthesized NPs offer a potent, environmentally friendly substitute for traditional biocides, especially in the prevention of biofilm-associated infections and water system biofouling.

### 3.6. Synergistic Effects with Phytochemicals

In an unusual way, GSNMs unite bioactive phytochemical coatings made from plant extracts with metallic cores. Phytochemicals present in GSNMs, such as flavonoids, alkaloids, terpenoids, and polyphenols, not only serve as natural reducing and capping agents but also significantly amplify the antimicrobial effects of the NPs [143]. Their contribution is realized through several interlinked mechanisms, as described below.

#### 3.6.1. Phytochemical-Induced Amplification of ROS Generation

When linked to the surface of NPs, phytochemicals such as flavonoids and phenolics facilitate electron transfer activities, which increase the formation of ROS. As a result, microbial cells experience increased oxidative stress [144].

#### 3.6.2. Metal Ion Chelation and Controlled Release by Phytochemical Ligands

Metal ions such as Ag^+^ or Zn^2+^ on the surface of NPs are bound by chelators produced from plants, such as tannins and catechins. This limits cytotoxicity and prolongs antimicrobial effectiveness by controlling their gradual, prolonged release [145].

#### 3.6.3. Membrane Disruption via Phytochemical–Lipid Interactions

Microbial membranes are destabilized by the integration of saponins and terpenoids in the phytochemical coating, which increases permeability to nanoparticle penetration [146].

#### 3.6.4. Phytochemical Inhibition of Resistance Pathways

By preventing bacterial resistance mechanisms like quorum sensing and efflux pump activity, some phytochemicals promote the accumulation of NPs and interfere with the formation of biofilms [147].

#### 3.6.5. Targeted Antimicrobial Binding Through Phytochemical Functional Groups

By improving the particular adherence of NPs to microbial surfaces, the functional groups found in phytochemicals (-OH, -COOH, and -CHO) maximize localized activity and reduce off-target effects [148].

#### 3.6.6. Stabilization and Biocompatibility Enhanced by Phytochemicals

The material is safer and more effective in aqueous disinfection conditions because the phytochemical shell improves colloidal stability and inhibits nanoparticle agglomeration [149].

Collectively, among GSNMs, AgNPs, ZnO NPs, and TiO_2_ NPs are the most reliable and widely studied. AgNPs show consistent, broad-spectrum activity against bacteria and fungi. Smaller particles (<20 nm) are generally the most effective. Their main mechanisms, ROS generation and membrane disruption, are well established, though the role of plant metabolites in boosting activity is still uncertain. ZnO NPs work through ROS production and zinc ion release, with stronger effects seen under UV or visible light and with smaller particle sizes. These trends are clear, but claims about added benefits from plant metabolites remain speculative. TiO_2_ NPs also rely on photocatalytic ROS generation. They are effective under UV light, less so in the dark. Efforts to enhance visible light activity, such as doping or composites, look promising but are not yet conclusive.

In short, AgNPs, ZnO, and TiO_2_ are the leading GSNMs with proven antimicrobial effects, but questions remain about the role of biological metabolites and their long-term stability in real-world use.

Table 4 enlists the most commonly reported practices in the synthesis, characterization, and antimicrobial testing of green-synthesized nanomaterials as summarized in this review. It provides a concise overview of how nanoparticles are typically produced using biological sources, how their physicochemical properties are assessed, and the standard assays applied to evaluate their antimicrobial activity.

## 4. Performance and Efficacy in Water Disinfection

The exceptional performance and effectiveness of GSNMs in water disinfection applications have attracted a lot of interest. They have potent antibacterial and antiviral properties due to their nanoscale size, increased surface reactivity, and the presence of bioactive phytochemicals produced via biological synthesis. These NPs are interesting candidates for sustainable water treatment technologies since they not only demonstrate broad-spectrum pathogen inactivation but also retain stability and efficacy in a variety of environmental circumstances [150].

### 4.1. Antibacterial and Antiviral Efficacy

**Figure 18:**
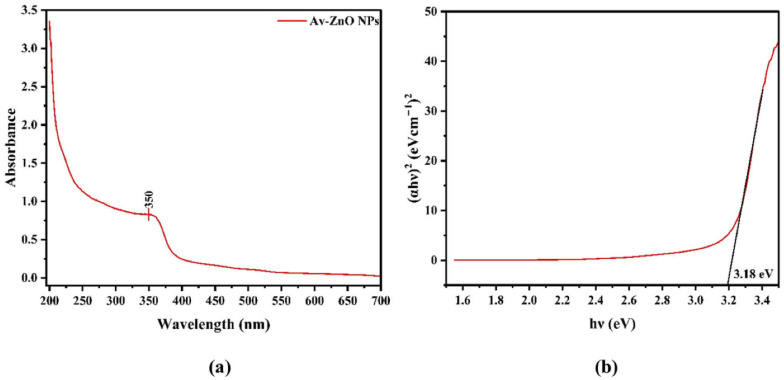
(**a**) Analysis of UV–vis absorption spectrum; (**b**) shows the band gap energy of Av–ZnO NPs synthesized from Aloe vera extract [99].

Green-synthesized NPs have been shown in numerous studies to have strong antiviral and antibacterial properties. Because of their strong oxidative potential and capacity to liberate Ag^+^ ions, silver NPs (AgNPs) are especially efficient. For instance, even at low concentrations of 10–20 µg/mL, AgNPs made from extracts of *Azadirachta indica* and *Camellia sinensis* reduced *Escherichia coli* and *Staphylococcus aureus* by >99.9% in 30 min [151]. In the same way, ZnO and CuO NPs produced by green synthesis demonstrate strong antiviral activity. With a reported viral titer reduction of >95% during UV-A exposure, ZnO NPs made with *Aloe vera* extracts successfully inactivated rotavirus through photocatalytic capsid breakdown [152]. By rupturing membranes and interfering with intracellular enzymatic activity, CuO NPs made from the peel extract of *Punica granatum* have shown comparable effects against both Gram-positive and Gram-negative bacteria [153]. By enhancing nanoparticle stability, charge interaction with microbial membranes, and ROS formation, the synergistic effect of biogenic capping agents such as polyphenols, flavonoids, terpenoids, and alkaloids improves efficacy [146]. The successful creation of ZnO NPs utilizing the green synthesis approach with *Aloe vera* plant extracts is confirmed by the unique absorption peak at 350 nm in the ZnO NPs spectra presented in Figure 18. ZnO NPs prepared by Aloe vera extract were subjected to UV-A at 365 nm [108], while TiO_2_ NPs prepared from algae extracts were used under natural sunlight [109].

This distinctive peak represents ZnO’s intrinsic band gap absorption, suggesting a high potential for photocatalysis in the presence of UV or near-UV light. Antibacterial and antiviral properties of ZnO NPs are greatly enhanced by the creation of reactive oxygen species (ROS), which are made possible by the effective generation of electron–hole pairs upon light absorption. The effectiveness of the plant-based synthesis approach in creating highly functional ZnO nanomaterials for water disinfection applications is thus demonstrated by the direct correlation between the confirmed structural and optical properties from the absorption spectrum and the capacity of NPs to inactivate bacteria and viruses by causing oxidative stress, rupturing viral capsids, and damaging microbial membranes [99].

### 4.2. Minimum Inhibitory Concentration (MIC) and Dose-Response

**Figure 19:**
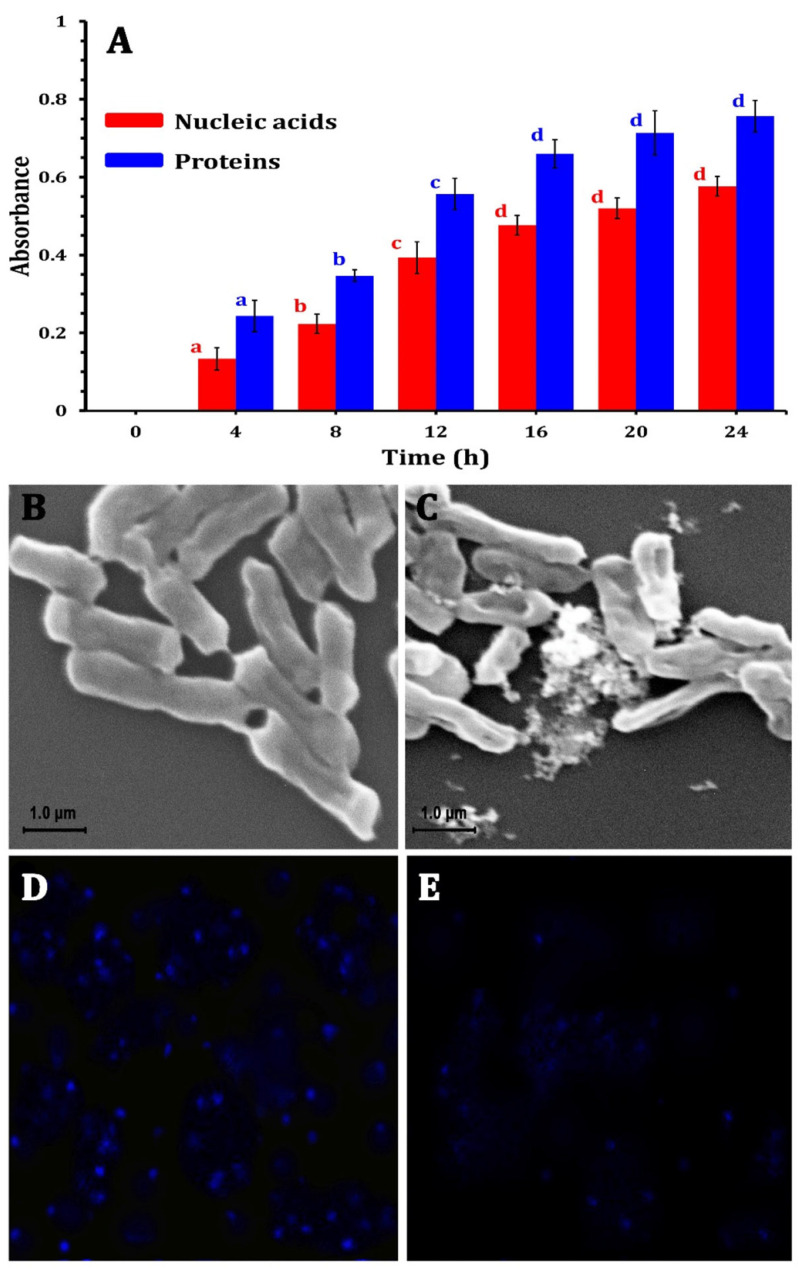
Mechanism of ZnO NPs against MDR *E. coli*: (**A**) protein and nucleic acid release; (**B**) SEM image of untreated cells; (**C**) SEM image after treatment; (**D**) DNA in untreated cells observed via confocal microscopy; (**E**) DNA changes in treated cells observed via confocal microscopy [155].

The effectiveness of GSNMs is frequently assessed using time-kill and Minimum Inhibitory Concentration (MIC) experiments. Mass concentration has frequently been utilized in numerous treatment investigations, especially in MIC and time-kill experiments. Some research papers analyze molar concentration to establish comparisons between metals or alloys, while in other cases, photocatalytic investigations involving ZnO and TiO_2_ standardize treatments per surface area to yield a more accurate representation of available active sites. This inconsistency prevents direct comparisons between the investigations. With full bactericidal action within 60 min, AgNPs made using *Moringa oleifera* extract demonstrated MIC values as low as 5 µg/mL against *Salmonella typhimurium* and *E. coli* [154]. ZnO NPs had robust bacteriostatic and bactericidal activities at doses ≥50 µg/mL, with dose-dependent inhibition zones spanning 10–25 mm against *Klebsiella pneumoniae* [155]. Figure 19A shows that ZnO NPs treatment resulted in a time-dependent increase in absorbance at 260 and 280 nm, suggesting protein and nucleic acid leakage. At 12 h, this leakage surged dramatically (*p* < 0.05), indicating that the MIC-level exposure weakened the membrane and caused vital biomolecules to flow out, which decreased cell viability.

At MIC doses, complementary SEM examination showed significant morphological damage. ZnO NPs-treated cells (Figure 19C) showed wrinkled, aggregated, and fractured membranes, indicating structural collapse caused by NPs, while untreated cells retained a smooth, undamaged surface (Figure 19B).

Furthermore, LSCM analysis revealed that treated E. coli cells had reduced DNA fluorescence (Figure 19E) in contrast to bright signals in controls (Figure 19D). This decrease suggests that ZnO NPs caused DNA damage, most likely as a result of reactive oxygen species (ROS)-induced oxidative stress. These actions strengthen the dose-dependent antibacterial efficacy seen in MIC experiments by interfering with important cellular functions like transcription and replication.

As a result, the capacity of ZnO NPs to harm bacterial membranes and genetic material is intimately associated with their dose-responsive antimicrobial action, demonstrating their effectiveness at MIC levels and bolstering their possible application in water disinfection. These results demonstrate the effectiveness of low-dosage green nanomaterials, which are beneficial for large-scale water treatment with little chance of secondary contamination or excessive nanoparticle exposure.

### 4.3. Biofilm Disruption Performance

Bacterial colonies covered in EPS form biofilms, which are extremely resistant to conventional disinfectants. Nevertheless, biofilm matrices can be efficiently penetrated and disrupted by GSNMs [156]. According to crystal violet staining and scanning electron microscopy (SEM), AgNPs made from *Ocimum sanctum* broke up well-established *Pseudomonas aeruginosa* and *Staphylococcus epidermidis* biofilms, resulting in a biomass reduction of more than 80% [157].

**Figure 20:**
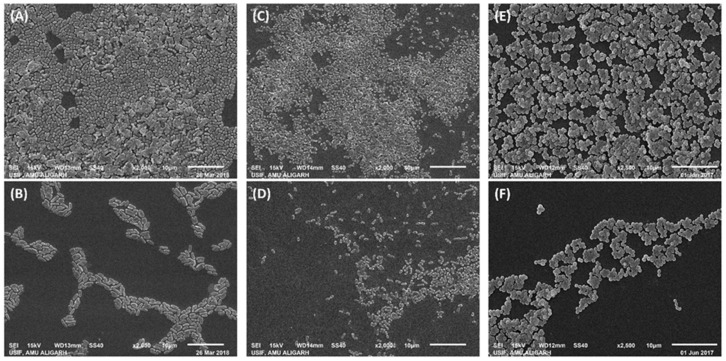
SEM images of biofilms formed by *S. aureus*, *P. aeruginosa*, and *E. coli*: (**A**,**C**,**E**) untreated; (**B**,**D**,**F**) treated ½ × MIC of ZnO NPs [158].

GSNMs are effective tools against biofouling in water systems because of the mechanism, which includes ROS generation, membrane destabilization, and interference with EPS synthesis pathways. Using scanning electron microscopy (SEM), which produced high-resolution pictures of the biofilm morphology, a thorough analysis of the biofilm structure after ZnO NPs treatment was conducted, illustrated in Figure 20. On glass coverslips, untreated *E. coli* cells developed dense, mature biofilms with smooth cell surfaces and significant embedding in EPS. On the other hand, bacterial adhesion and colonization were significantly decreased after treatment with ½ × MIC of ZnO NPs. A significant lack of EPS structures suggested that the integrity of the biofilm matrix had been compromised. This implies that early adhesion and biofilm formation can be successfully inhibited by even sub-inhibitory doses [158].

These findings demonstrate that ZnO NPs have antibiofilm activity that is dose-dependent and can efficiently break up biofilms at sub-lethal concentrations. For water disinfection systems, where biofilm control on surfaces is crucial to avoiding microbial regrowth and contamination, this makes them especially appealing. Their potential in integrated water treatment technologies is further highlighted by their broad-spectrum effectiveness against pathogens.

### 4.4. Efficacy in Real and Variable Water Conditions

The performance of NPs in real-life water matrices is a crucial component of assessing disinfection efficiency. Because natural organic matter (NOM), suspended solids, and fluctuating pH or turbidity can affect the stability and activity of NPs, laboratory efficacy may not always correspond to field effectiveness. The traditional pre-treatments, such as sedimentation, coarse filtration, and sand/activated carbon filtration, have been combined with GSNMs to manage turbidity and fouling produced by NOM. The processes of coagulation and flocculation using alum (Al_2_(SO_4_)_3_) in addition to natural coagulants have demonstrated the ability to enhance or support the antimicrobial efficacy of GSNM in both surface and wastewater situations [159].

According to Wang et al. [160], green-synthesized AgNPs demonstrated their resilience in moderately demanding environmental conditions by maintaining over 90% antibacterial efficacy even in river water samples with turbidity levels as high as 50 NTU. On the other hand, Li et al. [161] showed that surface-active compounds like fulvic and humic acids considerably reduced the disinfection effectiveness of ZnO NPs and CuO NPs in surface waters. These organic compounds were discovered to adsorb onto the surfaces of NPs, resulting in lower microbial inactivation rates, passivation, and ROS generation [162]. Table 5 summarizes the influence of NOM, turbidity, and matrix complexity on the disinfection efficacy of GSNMs across different real water sources.

These results emphasize the necessity of customized approaches to improve the efficacy of NPs in intricate aquatic systems. Performance in practical applications can be enhanced by adjusting the amount of NPs, the duration of contact, and perhaps adding pre-filtration or coagulation processes. Furthermore, functionalizing or altering the surface of NPs to prevent NOM fouling may present viable ways to preserve effectiveness in a variety of water sources.

### 4.5. Stability and Longevity of Efficacy

The durability and stability of nanoparticle action are important indicators of disinfection effectiveness. After being stored at ambient temperature for months, GSNMs made from plant or algae extracts have demonstrated long-term antimicrobial potency with little loss of action. Abebe et al. [166], for instance, showed that after 6 months of continuous use, AgNPs embedded in point-of-use ceramic filters maintained >90% disinfection efficiency.

The operational stability of NPs is further improved and leaching into treated water is avoided by encasing them in biopolymers such as chitosan or alginate or preventing them on support matrices such as membranes or activated carbon [167].

## 5. Applications in Water Disinfection

The impressive antimicrobial performance of GSNMs has enabled their integration into a variety of water treatment applications, ranging from small-scale domestic filters to advanced photocatalytic and hybrid systems. Their eco-friendly nature, biocompatibility, and scalability make them ideal candidates for sustainable water purification, particularly in resource-limited settings.

### 5.1. Nanomaterial-Embedded Filters and Membranes

GSNMs have been incorporated into water filtration membranes to enhance microbial removal. Ibrahim et al. [164] developed cellulose acetate membranes embedded with AgNPs synthesized using Moringa oleifera extract. These membranes showed significant improvement in bacterial inactivation while maintaining high water flux and mechanical strength. Similarly, Bhattacharya et al. [165] reported that ZnO NPs produced via green synthesis enhanced the performance of ceramic filters, extending their antimicrobial durability over prolonged use.

In another study, CuO and AgNPs embedded in polymeric membranes demonstrated synergistic effects in reducing biofouling and microbial colonization on membrane surfaces, offering potential for long-term use in decentralized water treatment systems [168].

In practical applications, GSNM-coated filters and membranes encounter issues of fouling and a progressive decline in disinfection efficacy due to the buildup of organic matter, biofilms, and inorganic scaling, which obstruct active sites and diminish water flux. Research indicates that the integration of nanomaterials (such as graphene or metal nanoparticles) improves hydrophilicity, antibacterial efficacy, and mechanical strength; nevertheless, foulants may obscure active surfaces, resulting in diminished performance over time [169]. Recent advancements include self-cleaning or regenerable methods, including electrocatalytic membranes that in situ decompose foulants and graphene-based composites that enhance antifouling resistance and provide more flux return post-cleaning [170]. The analysis underscores the necessity of validating operational stability under realistic settings, as elevated nanomaterial loading may lead to aggregation, pore blockage, or leaching, necessitating a careful equilibrium between durability and efficacy [171].

### 5.2. Point-of-Use and Household Water Treatment Devices

The low-cost and energy-efficient synthesis of GSNMs enables their application in point-of-use (POU) water treatment technologies, especially for rural and low-income communities. Gloria-Garza et al. [172] evaluated AgNP-coated filters synthesized from *Terminalia arjuna* and Tulsi extracts in rural India. These filters consistently achieved total coliform reduction to <1 CFU/100 mL, meeting WHO standards for drinking water.

Qubtia et al. [163] also demonstrated the use of plant-based AgNPs in gravity-fed household filtration units in Bangladesh. These filters maintained >95% bacterial removal efficiency over 6 months of continuous use, indicating strong potential for long-term, sustainable deployment.

### 5.3. Photocatalytic Water Disinfection Systems

Green-synthesized TiO_2_ and ZnO NPs have been used in solar photocatalytic disinfection (SPCD) systems. Blosi et al. [100] synthesized TiO_2_ NPs using algae (*Chlorella vulgaris*) and demonstrated their effectiveness under sunlight in degrading organic pollutants and inactivating *E. coli* and *Vibrio cholerae* in surface water samples. These systems are particularly attractive for off-grid water purification due to their reliance on natural sunlight and low operating costs.

### 5.4. Hybrid and Smart Water Treatment Systems

The integration of GSNMs with existing technologies has led to the development of hybrid systems. For example, combining AgNPs with activated carbon or sand filters improves both microbial and chemical contaminant removal. Some smart systems incorporate sensors to detect bacterial load and release NPs on demand, minimizing unnecessary exposure and preserving nanoparticle activity [173].

Such multi-functional systems offer enhanced efficacy, lower maintenance, and improved water quality, aligning with the goals of sustainable and circular water treatment strategies. GSNMs undergo a variety of changes in aquatic environments after fulfilling their disinfecting function, including oxidation, dissolution, aggregation, and sulfidation. These changes have a substantial impact on the stability, bioavailability, and toxicity of GSNMs. By reducing ion release following sulfidation, for example, these processes may lessen acute toxicity in some situations. However, they can also produce permanent forms that build up in sediments or interact with biota, which raises questions about long-term ecological effects. To guarantee reliable risk estimates, evaluations should, therefore, concentrate on both pristine GSNMs and their modified products, analyzing their environmental destiny, ion release behavior, and ecotoxicological impacts on aquatic systems [174,175].

## 6. Ecotoxicity and Environmental Safety

### 6.1. Cytotoxicity Concerns

Even when produced by environmentally sustainable ways, metal oxide and silver nanoparticles such as ZnO NPs and AgNPs can elicit the formation of reactive oxygen species (ROS) in non-target mammalian cells, leading to oxidative stress, apoptosis, and diminished viability. Green-synthesized ZnO nanoparticles derived from *Musa acuminata* leaf extract have shown dose- and time-dependent increases in reactive oxygen species (ROS) when exposed to Vero cells, which correlated with reduced cell viability and heightened apoptosis in comparison to chemically synthesized ZnO nanoparticles [176]. Likewise, green-synthesized AgNPs from *Euphorbia retusa* extract caused a decrease in mitochondrial membrane potential, elevated reactive oxygen species (ROS), and activated apoptotic markers (p53, Bax, caspase-3/9) in con-7 breast cancer cells that were developed at the Michigan Cancer Foundation (MCF), even at sub-cytotoxic concentrations [177]. Other green AgNPs produced using red beet (*Beta vulgaris*) root extract likewise significantly elevated ROS levels in non-cancerous human liver cells, leading to oxidative damage and apoptosis [178]. A thorough examination of green-synthesized AgNPs reaffirmed that ROS-mediated cytotoxicity encompassing lipid peroxidation, DNA damage, and membrane disruption serves as a primary mechanism across several human cell lines, albeit exhibiting decreased overall toxicity compared to chemically synthesized counterparts [179]. These data emphasize that whereas green production diminishes certain chemical residues, nanoparticle-induced reactive oxygen species continue to pose a significant risk to non-target cells. Consequently, comprehensive in vitro cytotoxicity assessments, meticulous ROS quantification, and surface modification or antioxidant capping techniques are imperative to guarantee safe utilization in applications like water purification and biomedical gadgets.

Table 6 provides a clear comparison between chemically synthesized nanoparticles (CSNPs) and green-synthesized nanoparticles (GSNPs) in terms of environmental destiny, ROS generation, cytotoxicity in mammalian cells, and antibacterial activity. This arrangement clearly delineates the differences and relative benefits between GSNPs and CSNPs for every type of nanoparticle.

The comparative study emphasizes that although GSNPs are generally more biocompatible and less cytotoxic than their CSNPs, this benefit is mostly due to the presence of bioorganic residues and natural capping agents that reduce unchecked ROS production. Long-term environmental effects are still a cause for concern, though. Although at lower levels than CSNPs, even GSNPs contribute to chronic toxicity, bioaccumulation, and possible food web transfer by releasing ions such as Zn^2+^ and Ag^+^ into aquatic ecosystems. Similar problems with partial dissolving and silt accumulation still exist for green-synthesized CuO and FeO_4_ NPs, although they show moderate reactivity and, in the case of FeO_4_, retrievability via magnetic separation. These results point to a significant trade-off. GSNPs offer better safety profiles without being intrinsically free from long-term environmental hazards, while CSNPs have greater antibacterial effectiveness but at the expense of significant ecological and human toxicity. Therefore, to confirm the environmental benefit of GSNPs over traditional methods, systematic long-term research on nanoparticle destiny, ion release kinetics, and chronic exposure pathways is crucial.

Long-term ecotoxicity and the environmental fate of GSNMs in aquatic environments remain important but still underexplored issues. Unlike short-term toxicity, chronic exposure can reveal how nanoparticles persist in water through processes such as slow dissolution, aggregation, and disaggregation cycles, and interactions with natural organic matter, all of which influence their bioavailability and toxicity [184]. For example, ZnO and CuO nanoparticles slowly release metal ions over time, causing sustained oxidative stress in algae and invertebrates, even at concentrations that are not immediately lethal [185]. Similarly, green-synthesized AgNPs, initially stabilized with biocompatible plant compounds, may undergo surface changes that increase Ag^+^ release, leading to long-term effects such as impaired growth, reproduction, and altered behavior in aquatic species like Daphnia magna and zebrafish [186]. The accumulation of nanoparticles in sediments also raises concerns about their transfer through the food chain and potential multigenerational impacts on benthic ecosystems. Environmental transformations such as photocatalysis, sulfidation, or complexation with natural ligands can further create new, ecotoxicologically active forms, making risk prediction challenging. These considerations highlight the need to go beyond simple acute toxicity tests, emphasizing long-term studies, mesocosm experiments, chronic exposure assessments, and life cycle analyses to fully understand the persistence, ecological risks, and bioaccumulation potential of green nanomaterials in aquatic ecosystems [187].

#### 6.1.1. In Vitro Evidence

Numerous human and microbial cell lines have been used to assess the in vitro cytotoxicity of GSNMs, especially metallic and metal oxide nanoparticles like molybdenum disulfide (MoS_2_), zinc oxide (ZnO NPs), copper oxide (CuO NPs), silver (AgNPs), and gold (AuNPs). To evaluate the cellular damage, metabolic disturbance, and loss of membrane integrity caused by nanoparticles, these investigations frequently employ assays such as MTT (3-(4,5-dimethylthiazol-2-yl)-2,5-diphenyltetrazolium bromide), LDH (lactate dehydrogenase) release, trypan blue exclusion, and ATP viability assays [188].

In human dermal fibroblast (HDF) and lung epithelial cells, for example, green-synthesized AuNPs have demonstrated modest to considerable cytotoxicity, with toxicity being dose- and time-dependent. It was suggested that the main mechanisms of cytotoxicity were membrane leakage, ROS production, and mitochondrial damage [189]. In a study by Al-Sarraj et al. [190], gold nanoparticles made with extract from *Salvia officinalis* showed significant toxicity at comparatively low concentrations, with IC_50_ values as low as 8.5 µg/mL in HepG2 cells. Also, with IC_50_ values of around 981 µg/mL, green-synthesized Ag_2_O/AgNPs made with Pueraria lobata root extract demonstrated potent cytotoxic effects in human keratinocyte (HaCaT) cells. The nanoparticles showed decreased biocompatibility despite having strong antibacterial and antioxidant qualities; this could be because of oxidative stress mechanisms or unregulated ion release [191].

**Figure 21:**
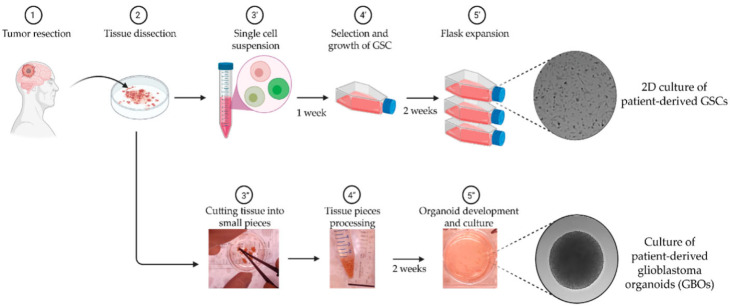
In vitro modeling of glioblastoma tumor microenvironment [192].

Figure 21 demonstrates how in vitro platforms can simulate complex tumor biology. By incorporating cellular and extracellular elements of the glioblastoma microenvironment, such models enable researchers to investigate cancer cell behavior and assess nanoparticle-based or chemotherapeutic treatments in a reproducible and ethical manner [192].

On the other hand, certain nanomaterials have demonstrated positive characteristics of in vitro biocompatibility. For instance, even at concentrations as high as 250 µg/mL, MoS_2_ nanoflowers that were made in an environmentally friendly manner and capped with L-cysteine showed over 90% viability in human foreskin fibroblast (HFF) cells and were still successful in suppressing bacterial pathogens [193]. The natural amino acid capping agent and the multilayer structure, which reduced oxidative stress and membrane damage, were credited with the harmless behavior [194].

One of the GNMs that has been investigated the most, ZnO NPs, has cytotoxic characteristics that vary greatly [195]. At doses above 40 µg/mL, for example, ZnO NPs made using *Azadirachta indica (neem)* extract demonstrated moderate cytotoxicity toward A549 human lung cancer cells, with enhanced ROS production and notable cell membrane rupture [196]. However, ZnO NPs made using green tea (*Camellia sinensis*) showed less cytotoxicity against L929 fibroblast cells, maybe as a result of capping molecules rich in polyphenols that improved cell compatibility [197].

Furthermore, HeLa and HEK-293 cells were used to test CuO NPs made with *Moringa oleifera* extract; the IC_50_ values were 29 µg/mL and 43 µg/mL, respectively. At greater concentrations, the nanoparticles resulted in DNA damage, lipid peroxidation, and mitochondrial fragmentation [198].

**Figure 22:**
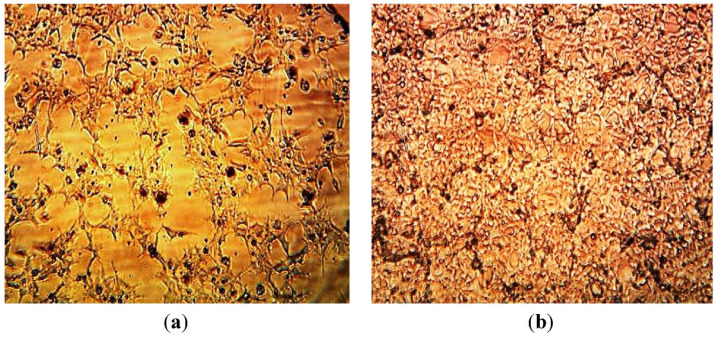
Time-dependent morphological changes in HEK 293 cells exposed to green-synthesized silver nanoparticles (**a**) 6 h after injection of 4 ppm AgNPs reduced using Eucalyptus extract; (**b**) 48 h after injection of 4 ppm AgNPs. [201].

Many variables, including nanoparticle size, shape, surface charge, capping agent, and composition, affect the variation in in vitro cytotoxicity of GNMs. For instance, cytotoxic effects are enhanced by smaller particles with a larger surface area because they are more likely to generate ROS and pass through cell membranes [199]. Furthermore, depending on their chemical makeup, the capping biomolecule made from plant extracts can either lessen or increase cellular stress responses, which is a critical factor in modulating toxicity [200]. Figure 22 illustrates the in vitro biocompatibility and cytotoxic dynamics of green-synthesized AgNPs in human embryonic kidney (HEK 293) cells. While early exposure (6 h) shows minimal damage, extended incubation leads to reduced confluency and cellular integrity, indicating a time- and dose-dependent cytotoxic profile consistent with nanoparticle-induced oxidative stress and membrane interaction [201]. Early exposure causes minimal disruption, but prolonged contact leads to significant morphological alterations, indicating a gradual buildup of oxidative stress, membrane damage, or apoptosis. These findings underscore the importance of dose and exposure time when evaluating the biosafety of nanomaterials intended for water disinfection. These results emphasize how crucial it is to modify nanoparticle manufacturing techniques for certain environmental and medicinal uses [172]. Although a large number of GNMs show encouraging antimicrobial activity, their lethal effects on non-target mammalian cells highlight the necessity of thorough toxicological testing prior to environmental deployment.

#### 6.1.2. In Vivo Evidence

Even though in vitro research offers important information about the cytotoxic potential of GNMs, it is unable to accurately replicate the intricate biological interactions found in living beings. Therefore, even though they are less common, in vivo studies are crucial for evaluating organ-specific toxicity, systemic reactions, biodistribution, and clearance processes. According to current in vivo studies, GNMs can exhibit acceptable tolerability and, in certain situations, even medicinal potential when appropriately tailored [202].

In one noteworthy study by Hassan [203], gambogic acid (GA)-capped ZnO nanoparticles were greenly synthesized and then loaded with aspirin (Asp) to create a multifunctional anticancer formulation. When given to animals with Ehrlich ascites carcinoma (EAC), this nanoformulation dramatically increased anticancer efficacy in comparison to controls. More significantly, the treatment reduced systemic toxicity: serum biomarkers of liver function (e.g., AST, ALT) were significantly decreased, while hematologic parameters, including hemoglobin concentration and total WBC count, were returned to nearly normal levels. Liver samples examined histopathologically showed maintained hepatic architecture, suggesting little damage to the organs [204]. This example shows that biocompatible green-synthesized ZnO NPs, particularly when functionalized with phytochemicals, can exert selective cytotoxicity toward diseased tissue while minimizing injury to healthy organs, despite the fact that it focuses on drug delivery rather than disinfection [205]. The mechanisms that may also be involved in antibacterial applications; tumor microenvironment sensitivity, surface charge interactions, and regulated ion release are most likely responsible for this selective activity [206].

Likewise, mice were given sub-lethal intraperitoneal injections of green-synthesized gold nanoparticles (AuNPs) enhanced with extract from *Pelargonium graveolens* for a duration of 14 days. Liver enzyme levels (ALT, AST) and kidney function indicators (urea, creatinine) did not significantly differ in the treated group, and organ histology showed normal cytoarchitecture. But a small amount of Au buildup was seen in the liver and spleen, suggesting that long-term clearance investigations are necessary [207].

**Figure 23:**
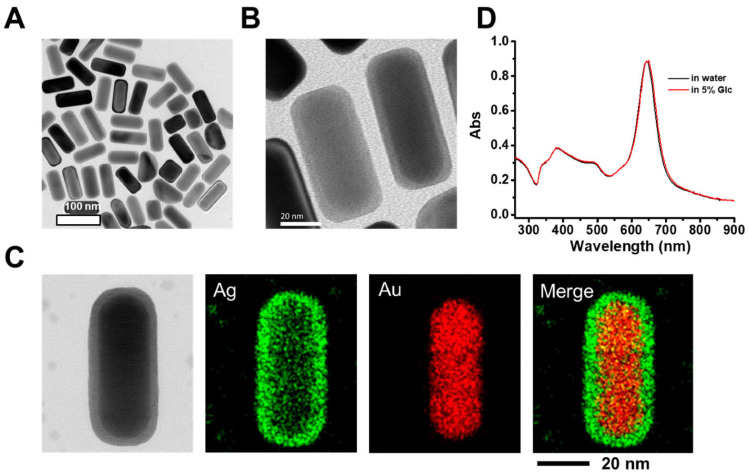
Transmission electron microscopy and elemental mapping of Au@Ag NRs in rat tissue (**A**,**B**) Typical TEM images of Au@Ag NRs; (**C**) TEM image of an individual Au@Ag NR and its corresponding EDS (energy disperse spectroscopy) element maps of Au, Ag, and their overlay, respectively; (**D**) UV-vis-NIR (ultraviolet-visible-near infrared ray) extinction spectra of Au@Ag NRs dispersed in water and 5% glucose solution, respectively [208].

Figure 23 presents TEM images alongside Energy-dispersive X-ray spectroscopy (EDS) elemental maps that reveal the shape and distribution of gold-core silver-shell nanorods (Au@Ag NRs) within rat tissues following 28 days of oral administration. The distinct visualization of gold and silver confirms the nanoparticles’ presence and precise localization. Combining TEM with elemental mapping offers clear in vivo evidence of nanoparticle uptake and distribution at the cellular level, which is vital for assessing their behavior, safety, and potential biomedical applications [208].

In an updated in vivo investigation, Thejaswini et al. [209] used phyto-mediated TiO_2_ nanoparticles, which were made with extract from *Ocimum sanctum*, to assess the safety and effectiveness of disinfection in a mouse model. After 30 days of intake, the NPs were added to drinking water, and no anomalies in the liver or kidney tissue were seen. The fact that blood biochemical markers stayed within reference ranges supports the fact that the nanomaterials used in water purification settings are non-toxic [210].

Although these encouraging results, there are still a number of significant gaps in the wider use of green-synthesized NPs for water disinfection from the perspective of in vivo safety. Notably, there is still a lack of adequate characterization about systemic biodistribution, accumulation, clearance pathways, and chronic exposure outcomes [211]. Uncertain long-term effects can result from the accumulation of nanoparticles, particularly metallic ones, in the kidneys, lungs, liver, and spleen. Although plant-derived capping agents introduce compositional heterogeneity that may impact biological interactions, green synthesis may offer greater biocompatibility [212]. Furthermore, the behavior of nanoparticles in physiological fluids (such as agglomeration, the formation of protein corona, and surface modifications) can vary greatly from that in vitro, underscoring the need for thorough pharmacokinetic and toxicokinetic investigations designed especially for environmental applications like water disinfection [213].

All things considered, our results lend credence to the idea that GNMs can demonstrate in vivo tolerability and perhaps medicinal benefit, especially when they are well formed and biologically capped. However, before being widely used in actual water treatment applications, standardized in vivo toxicity assessment procedures that are in line with Organisation for Economic Cooperation and Development [OECD] criteria are crucial due to the novelty and diversity of green synthesis pathways [214].

#### 6.1.3. Non-Target Effects

Green-synthesized nanoparticles (NPs) can have unexpected effects on aquatic creatures that are not their original target, including algae, invertebrates, and beneficial bacteria, even if they are produced using environmentally favorable techniques. The intrinsic surface reactivity, ion release, and ROS formation linked to metallic and metal oxide nanoparticles are the main causes of these effects [215].

According to a number of ecotoxicity studies, AgNPs and ZnO NPs can impede aquatic species’ mobility, induce oxidative stress, and restrict growth even when they are made from plant extracts. For example, at concentrations above 30–50 µg/mL, Artemia salina and Daphnia magna exposed to green AgNPs demonstrated decreased survival and movement [216]. Similarly, green ZnO NPs hindered photosynthetic efficiency and damaged chloroplast integrity in freshwater algae like Chlorella vulgaris [217]

**Figure 24:**
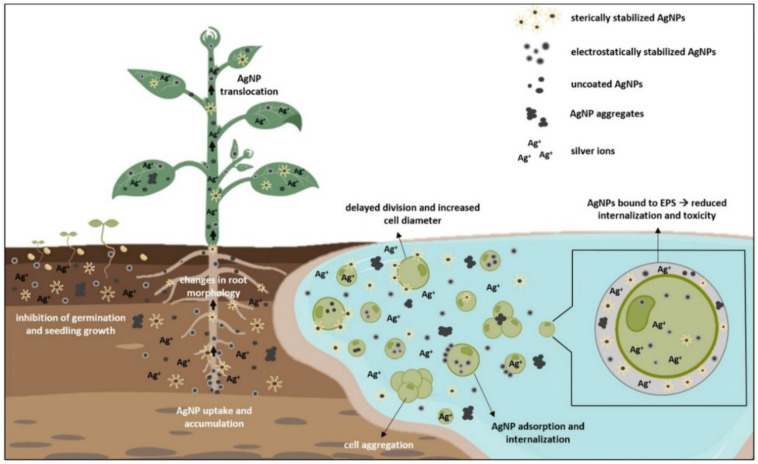
Interaction of AgNPs with *Chlorella vulgaris* showing ROS generation, enzymatic inhibition, and chloroplast damage, indicating non-target toxicity [218].

Figure 24 illustrates the mechanism by which silver nanoparticles (AgNPs) exert toxic effects on non-target algal species like *Chlorella vulgaris*. It visually demonstrates how GSNMs, particularly AgNPs, impact non-target aquatic organisms like algae (*Chlorella vulgaris*), which are not the intended microbial targets of water disinfection. Upon exposure, AgNPs disrupt cell membrane integrity and trigger excessive reactive oxygen species (ROS) generation. This oxidative stress overwhelms the algal antioxidant defense system (including catalase, superoxide dismutase, and peroxidase), leading to enzymatic inhibition, chloroplast damage, and decreased photosynthetic efficiency [218]. Such effects are central to understanding the ecological risks of green-synthesized nanoparticles in aquatic environments.

Biogenic NPs have the ability to bind to microbial membranes, disrupt enzymatic processes, and cause immunological or genotoxic reactions even at sublethal concentrations. These effects are explained by the nanoparticles’ tiny size, large surface area, and capacity to change the cellular redox balance and pass through biological barriers [219]

Green production does not remove the bioactivity of nanoparticles, but it does avoid hazardous chemical residues. Thus, extensive ecological risk assessment, including long-term exposure studies, effects on microbial communities, and accumulation in food chains, is required before such NPs are widely used in water treatment systems.

### 6.2. Environmental Safety Concerns

Green-synthesized nanomaterials (GSNMs) are increasingly acknowledged as sustainable and efficient agents for water disinfection, attributed to their environmentally friendly synthesis methods and potent antimicrobial characteristics. Nonetheless, despite their eco-friendly origins, apprehensions about their environmental safety and possible toxicity are being increasingly expressed. Comprehending these hazards is essential to guarantee that the utilization of GSNMs does not unintentionally jeopardize aquatic habitats or human wellbeing.

The Organization for Economic Cooperation and Development (OECD) has acknowledged the necessity to modify current testing protocols to accommodate the distinctive characteristics of nanomaterials. OECD Test Guideline No. 125 delineates precise procedures for assessing the particle size and size distribution of nanomaterials, including their nanoscale dimensions and behavior in diverse environments. The OECD has produced Guidance Document No. 317, which provides recommendations for evaluating the environmental destiny and ecotoxicity of nanomaterials, highlighting the necessity of accounting for their particle characteristics in assessments of aquatic and sediment toxicity [220,221].

The International Organization for Standardization (ISO) has established standards to enhance the characterization and testing of nanomaterials. This delineates the criteria for identifying measurands to characterize nano-objects and their agglomerates and aggregates, establishing a framework for uniform measurement techniques [222]. Additionally, another document provides directives for the biological assessment of medical devices incorporating nanomaterials, emphasizing the necessity for specialized testing protocols to evaluate the biocompatibility of devices based on nanomaterials [223].

These changes seek to guarantee that testing procedures are appropriate for the unique properties of nanomaterials, thereby improving the reliability and pertinence of safety evaluations in regulatory frameworks.

#### 6.2.1. Bioaccumulation

Persistent hydrophobic substances can accumulate in aquatic species through many mechanisms: direct uptake from water via gills or skin (bioconcentration), ingestion of suspended particles, and consumption of contaminated food (biomagnification). Despite the absence of observable acute or chronic effects in typical ecotoxicity assessments, bioaccumulation must be considered a hazard criterion [224]. Notwithstanding the environmentally friendly allure of green-synthesized nanoparticles (NPs), research indicates that they may nonetheless bioaccumulate in aquatic species and present ecological hazards. Govahi et al. [225] noted considerable zinc accumulation in the liver and muscle tissues of *Cyprinus carpio* exposed to zinc oxide nanoparticles (ZnO NPs) derived from *Scrophularia striata* extract, although these green ZnO NPs caused less physiological stress compared to their chemically synthesized equivalents. A.H. Mandal et al. [226] examined the effects of ZnO nanoparticles on fish and their feeding species, confirming their bioaccumulation and sub-lethal affects throughout trophic levels. Silver nanoparticles (AgNPs), extensively utilized for their antibacterial characteristics, additionally demonstrate bioaccumulation and trophic transfer within aquatic habitats. Lazim et al. [227] documented the extensive presence of Ag and AgNPs in rivers, with measurable concentrations in sediment, aquatic flora, and fish tissues, signifying trophic transfer. Liang et al.’s [228] experimental investigations further illustrated the impact of organic matter on the bioavailability and accumulation of AgNP within a food chain consisting of algae, zooplankton, and fish. Additionally, RSC researchers have documented bioconcentration factors above 1000 in fish after exposure to AgNP, substantiating considerable trophic transfer potential [229]. Another environmental assessment validated similar findings, emphasizing how modifications of nanoparticles (e.g., sulfidation, aggregation) influence uptake and retention in aquatic organisms [230]. Producing long-term bioaccumulation and chronic exposure data for nanomaterials is crucial for regulatory decision-making. Standardized testing protocols and metadata reporting systems facilitate systematic data collection, while cooperation among community groups, academic researchers, and regulators prioritizes pertinent endpoints and ensures data integrity. The integration of community initiatives with scientific research facilitates thorough monitoring of nanomaterials in the environment, hence aiding risk assessment and regulatory measures [231,232,233].

#### 6.2.2. Environmental Persistence

GSNMs, including silver nanoparticles (AgNPs), zinc oxide (ZnO) nanoparticles, and cerium oxide (CeO_2_) or analogous metal oxides, exhibit extended persistence in environmental compartments owing to gradual transformations and possible bioaccumulation. The utilization of green-synthesized zinc oxide nanoparticles (GSZnONPs) markedly diminished the levels of chromium (Cr), copper (Cu), and zinc (Zn) in the overlaying and pore water of fishpond sediments. This lowering not only diminishes the leachability of certain heavy metals but also significantly reduces their bioavailability [234]. Green-synthesized CuO nanoparticles derived from plant extracts (e.g., *Parthenium hysterophorus*) exhibit structural stability and low solubility, allowing them to remain in aquatic settings following remedial treatments [235]. Biosynthesized ZnO nanoparticles utilizing *Moringa oleifera* leaf extract provide crystalline particles (~5–40 nm) with stable surface charges, indicating minimal natural degradation and prolonged environmental persistence [236]. Moreover, comprehensive evaluations of green or eco-synthesized metal oxide nanoparticles reveal that their environmental behavior, comprising aggregation, surface alterations, and trophic transfer, is insufficiently defined, emphasizing a potential risk of persistence and biomagnification contingent on particle chemistry and ecosystem context [237].

#### 6.2.3. Regulatory Gaps

The swift expansion of GSNMs for environmental and biological applications has revealed significant regulatory deficiencies and variations in toxicity evaluation systems. Standard OECD Test Guidelines (TGs) and Guidance Documents (GDs), formulated for conventional chemicals, frequently fall short for nanomaterials due to their distinct physicochemical properties, such as aggregation, dissolution kinetics, and particle-specific dosage metrics in testing environments. The OECD’s 2021 guidance (Document 317) seeks to modify aquatic toxicity testing for nanomaterials by addressing dispersion protocols, alternative dose metrics, and exposure monitoring; however, it recognizes ongoing difficulties in attaining inter-laboratory reproducibility and dependable hazard classification [220]. A 2023 workshop report on current genotoxicity test guidelines highlights shortcomings in existing testing methodologies, lacking consensus on addressing nano-specific exposure conditions, cellular internalization, and physicochemical characterization criteria. The paper underscores the pressing necessity for novel approach methods (NAMs) and elucidation of their regulatory application in evaluating the genotoxic potential of nanomaterials [238]. The slow rate of standardization exacerbates the situation. The 2024 roadmap document indicates that, although several OECD and ISO standards now incorporate nano-relevant methodologies, the coverage of endpoints is still inadequate, particularly for novel materials such as advanced graphene derivatives and polymer nanocomposites [239]. Efforts for further regulatory harmonization, exemplified by the Malta Initiative and Nano Harmony, are underway but remain well short of achieving a mature worldwide agreement [240]. Furthermore, authoritative evaluations of nanotoxicity research indicate that existing toxicity testing frequently depends on ad hoc or laboratory-specific protocols, resulting in inconsistencies in sample preparation, dosage metrics, and exposure duration, thereby rendering comparative risk assessments unreliable and hindering regulatory decision-making [241]. The absence of harmonized assay cascades, validated methods, and mutual acceptance frameworks restricts the safe and standardized deployment of nanomaterials due to regulatory ambiguity.

### 6.3. Essential Studies Before Large-Scale Deployment

Prior to increasing the production of green-synthesized nanomaterials, comprehensive risk assessment models, dose optimization, and degradation studies are important to guarantee safety and regulatory adherence. Despite originating from biological precursors, the physicochemical properties of nanoparticles such as size, surface charge, morphology, and aggregation can still provoke oxidative stress, neurotoxicity, or ecological damage, particularly at elevated exposure levels, as evidenced by studies on green-synthesized AgNPs in rodent brains, which reveal dose-dependent inhibition of acetylcholinesterase and alterations in neurotransmitters [242]. Computational risk assessment techniques, such as QSTR/QSAR modeling of nanoparticle-bio interactions, facilitate toxicity predictions based on structural descriptors and aid in hazard identification without extensive in vivo testing [243]. Dose optimization using in vitro and in vivo testing determines the least effective antibacterial or therapeutic doses while reducing undesired cytotoxicity or environmental buildup. Furthermore, degradation and destiny studies evaluating persistence, surface transformation, dissolution, or ligand degradation are essential for forecasting the behavior of nanomaterials over time in biological or environmental matrices, thus averting long-term detrimental impacts [244]. Collectively, these measures establish a scientifically sound basis for the responsible implementation of green nanosystems in practical environments.

Recent life cycle evaluations (LCAs) contrasting green and conventional nanoparticle manufacturing methods underscore substantial environmental implications. Rodríguez-Rojas et al. [245] performed a study on the LCA of titanium dioxide (TiO_2_) nanoparticles manufactured via Cymbopogon citratus leaf extract and the traditional chloride method. The results demonstrated that although the green synthesis approach minimized the utilization of hazardous chemicals, it nonetheless contributed to greenhouse gas emissions and respiratory issues due to energy-intensive processes such as calcination and drying. Conversely, the traditional chloride method demonstrated elevated emissions linked to chemical reagents. A separate review evaluated the environmental ramifications of biogenic compared to chemical synthesis of silver (Ag) and gold (Au) nanoparticles, indicating that although biogenic methods circumvent toxic chemical reductants, they may result in elevated non-product metal outputs, specifically 27–37% for Au and 46.7% for Ag, thereby exacerbating environmental burdens unless recovery systems are established [246]. Kirubakaran et al. [247] emphasized that although the synthesis of green nanoparticles diminishes dependence on toxic chemicals, it frequently necessitates energy-demanding post-synthesis processes and produces biomass waste, highlighting the necessity for thorough life cycle assessments to assess environmental repercussions.

## 7. Challenges and Future Prospects

**Figure 25:**
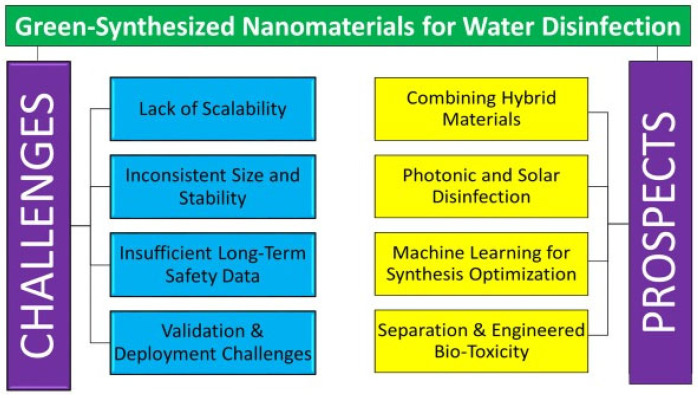
Challenges and future prospects of GSNMs.

GSNMs provide environmentally sustainable alternatives for diverse applications; yet, they encounter obstacles including variable synthesis processes, restricted scalability, and ambiguous environmental and toxicological effects. Despite these challenges, future prospects appear optimistic due to breakthroughs in green chemistry, enhanced process control via artificial intelligence, and increasing interest in safe, sustainable nanomaterials for applications in health, agriculture, and environmental remediation. Figure 25 highlights different challenges and prospects of GSNMs for water disinfection applications.

### 7.1. Challenges

Nanomaterials created by green methods, including environmentally benign biological agents such as plant extracts, microorganisms, and biopolymers, present a sustainable alternative to traditional chemical synthesis. Although these materials have potential for diminishing environmental toxicity and facilitating safer biomedical, agricultural, and industrial applications, they encounter numerous significant obstacles. Critical concerns encompass restricted regulation of particle size and shape, inconsistency in biological reducing agents, challenges in scalability, absence of established techniques, and inadequate comprehension of their prolonged environmental and biological interactions. Moreover, guaranteeing reproducibility, stability, and adherence to regulations further hinders their extensive use.

#### 7.1.1. Lack of Scalability in Green Synthesis

Numerous plant resources are utilized for the green production of nanoparticles, and several researchers have investigated locally available and abundant plant species. These discoveries enable the comprehensive utilization of indigenous plants; yet, achieving extensive worldwide production of green-synthesized nanoscale metals remains challenging. Plants utilized for the synthesis of silver nanoparticles (Ag NPs) include coconut, predominantly located in the Philippines, Malaysia, Sri Lanka, India, and China [248], as well as Acacia, primarily found in Arabia, Africa, and mainland China [249]. The production of Cu nanoparticles utilizes Andean blackberry (*Rubus glucus* Benth.), mostly found in Ecuador, Colombia, and the Andean region of Central and South America [250]. Moreover, certain raw materials are classified as secondary products requiring additional processing, hence increasing both complexity and cost in the technology prior to their application in the green synthesis of nanoscale metals. Tea extract serves as an illustration. Wang et al. [251] directly introduced pure tea polyphenols to synthesize NZVI; however, the extraction and purification processes were not economically viable.

#### 7.1.2. Inconsistent Nanoparticle Size and Stability

The dimensions and morphology of nanoparticles produced by various extracts exhibit significant variability, and the assessed properties are inadequate. Gao et al. [252] found the particle size of NZVI nanoparticles generated from grape seeds ranged from 63 to 381 nm, but Chahardoli et al. [253] observed the size of silver nanoparticles produced from *Nigella arvensis* leaves varied from 5 to 100 nm. The manufactured NZVIs utilizing Nettle and Thyme leaf extracts appear to form irregular clusters [254], whereas the Pd NPs produced from *Colocasia esculenta* leaf extract similarly exhibit irregular forms [255]. The particle size of Au nanoparticles produced from aqueous *Elaeis guineensis* (oil palm) and *Galaxaura elongata* exhibited significant variation [256,257]. GSNMs frequently experience instability and inadequate reproducibility, principally attributable to the intrinsic unpredictability of biological reducing and capping agents. Variables like plant species, extract content, pH, temperature, and reaction duration can result in substantial batch-to-batch variations in nanoparticle size, morphology, surface chemistry, and colloidal stability [258]. This discrepancy compromises product uniformity and may hinder functional performance in practical applications. Additionally, contaminants and free radicals in biological extracts might compromise the stability of nanomaterials over time, leading to aggregation or diminished activity during storage [259]. This unpredictability hinders the standardization of techniques, scaling of production, and maintenance of consistent quality control, presenting significant difficulties to the economic viability of green nanomaterials.

#### 7.1.3. Insufficient Long-Term Safety Data

Despite the green synthesis of nanomaterials reducing the reliance on toxic chemicals, a considerable deficiency in long-term safety data persists, especially concerning chronic exposure, persistence, and bioaccumulation in biological and environmental systems. Most current research emphasizes acute or subchronic trials (ranging from days to a few months), resulting in inadequate exploration of long-term consequences, including tissue accumulation, chronic inflammation, or genotoxicity [260]. Single-dose investigations of silica nanoparticles in mice demonstrated organ-level retention and chronic inflammatory effects persisting for up to one year post-exposure [261]. Likewise, green-synthesized AgNPs delivered to rats over several weeks elicited dose-dependent neurotoxicity and oxidative stress markers in brain tissue, highlighting possible long-term neurological hazards [242]. In fish models, extended exposure to biosynthesized AgNPs led to bioaccumulation and injury to gill tissue, indicating issues regarding ecological persistence and trophic transmission [262]. Green nanoparticles may induce genotoxic effects with prolonged exposure, even at concentrations considered mildly harmful in short-term evaluations [263]. These constraints underscore the necessity for extensive, meticulously designed studies spanning many months to years to assess biodegradation, biodistribution, immunological response, and long-term safety prior to large-scale implementation.

Despite the encouraging laboratory performance of green-synthesized nanomaterials, a substantial gap exists in proving their efficacy in real-world scenarios, particularly under diverse environmental and operating settings. A multitude of studies are restricted to controlled laboratory environments, resulting in ambiguities regarding the impact of variables such as local pH, temperature, organic or metallic pollutants, and soil or water matrices on nanoparticle behavior, stability, activity, and recovery in field applications [264]. In soil and water remediation, the effectiveness and processes of green-synthesized bimetallic nanoparticles exhibit significant regional variability due to environmental heterogeneity, raising concerns regarding their consistency and unexpected effects on non-target organisms [265]. Although there have been successful pilot applications in controlled reactors or greenhouse systems, there is a scarcity of studies on comprehensive field-scale testing or lifecycle assessments that replicate real operational settings [244]. Moreover, in the absence of long-term performance monitoring, there is a restricted understanding of durability, possibility for reuse, regeneration, and environmental impact over time [266]. These constraints highlight the urgent need for comprehensive real-world trials, encompassing pilot or on-site deployment investigations, prior to the dependable integration of green nanomaterials into environmental, agricultural, or industrial applications.

### 7.2. Future Directions

GSNMs exhibit significant potential for sustainable applications; nevertheless, further research is required to improve their efficacy. Essential directives encompass the advancement of hybrid nanomaterials, solar-powered disinfection systems, and portable filtration devices. Machine learning can enhance synthesis, while advanced separation techniques, toxicity assessments, and genetically modified microorganisms will provide safer, scalable production.

#### 7.2.1. Hybrid Materials Combining Multiple Nanoparticles

The synthesis of hybrid nanomaterials through the amalgamation of two or more green-synthesized nanoparticles is an innovative approach to augment multifunctionality. For example, ZnO/Ag_2_O and ZnO/SnO_2_ nanocomposites, manufactured by environmentally friendly processes, demonstrate synergistic effects that enhance photocatalytic degradation, antimicrobial efficacy, and electrochemical characteristics [267,268]. These hybrid systems leverage heterojunction formation, which improves charge separation and augments redox reactivity under visible light. Future research should focus on optimizing biosynthetic conditions, enhancing interfacial bonding across components, and improving the scalability of these hybrid systems for applications in water remediation, sensing, and energy storage.

#### 7.2.2. Light-Activated and Solar-Powered Disinfection Systems

GSNMs like ZnO, TiO_2_, and doped oxides exhibit significant potential for solar-powered disinfection owing to their capacity to produce reactive oxygen species when exposed to sunshine. Light-activated systems employing green-synthesized nanoparticles have demonstrated considerable efficacy in decomposing organic dyes and pathogens in wastewater [267,269,270]. These materials can be included in portable solar reactors or coatings for decentralized water filtration in off-grid and rural regions. Future development must prioritize the enhancement of visible-light sensitivity and the augmentation of long-term photocatalytic durability under variable outside settings.

#### 7.2.3. Machine Learning for Synthesis Optimization

The intricacy and diversity of green synthesis methods frequently lead to variable nanoparticle quality. Incorporating machine learning (ML) into the synthesis pipeline can resolve this issue by forecasting optimal conditions for nanoparticle formation based on input factors such as pH, temperature, and precursor concentration. Recent studies have shown the efficacy of machine learning methods, such as random forests and XGBoost, in predicting the size, shape, and functioning of nanomaterials, thereby enhancing repeatability and diminishing experimental effort [271,272]. Future studies may investigate real-time machine learning feedback systems integrated with robotic synthesis platforms to completely automate and enhance green nanoparticle creation.

#### 7.2.4. Development of GSNM-Integrated Portable Filters

Portable filtration systems integrated with GSNMs have garnered interest for their capacity to merge physical filtration with antimicrobial and adsorptive capabilities. Silver nanoparticles produced through eco-friendly methods have been effectively integrated into cellulose-based membranes, markedly enhancing microbial inactivation and heavy metal elimination [273,274]. These filters are especially designed for point-of-use water purification in disaster-stricken areas or economically disadvantaged regions. Future development must prioritize mechanical durability, regenerative ease, and sustained field performance under diverse environmental circumstances.

#### 7.2.5. Separation, Toxicity Studies, and Genetically Engineered Biosystems

Notwithstanding the environmental advantages of green synthesis, difficulties persist in effectively isolating nanoparticles from biomass without jeopardizing product stability. Moreover, there exists an urgent necessity for thorough toxicological assessment of GSNMs across biological systems to evaluate long-term hazards [275,276]. Recent studies are investigating the application of genetically modified microorganisms (GMMs) to augment nanoparticle synthesis and metal absorption, hence enhancing yield consistency and functional stability [277]. GMMs can be customized to generate specific enzymes or biomolecules, providing a progressive, scalable method for the manufacture of green nanoparticles.

## 8. Conclusions

GSNMs are emerging as a powerful and eco-friendly option for antimicrobial water treatment. By using plants, microbes, algae, and natural polymers as reducing and stabilizing agents, they merge the strengths of nanotechnology with the principles of green chemistry. Among them, silver, zinc oxide, and titanium dioxide nanoparticles stand out for their consistent and broad-spectrum antimicrobial effects, which arise from multiple mechanisms such as membrane disruption, ROS generation, ion release, DNA damage, and biofilm inhibition.

Despite these advantages, there are clear limitations. Different biological sources and synthesis methods often lead to inconsistent results, making reproducibility a challenge. While lab studies strongly support their antimicrobial potential, uncertainties remain around toxicity, long-term stability, and environmental fate when these materials are used in real-world conditions. The exact role of phytochemicals and other natural capping agents in shaping nanoparticle activity also remains only partly understood.

Moreover, progress will depend on standardizing synthesis protocols, improving scalability, and ensuring that safety is thoroughly tested through systematic studies. Research on nanoparticle recovery, reuse, and risk management will also be crucial to prevent unintended environmental impacts. If these gaps can be addressed, GSNMs could play a vital role in building safer and more sustainable water disinfection technologies.

## Figures and Tables

**Table 1:** Objective comparison of greenness metrics for GSNM synthesis techniques.

Metric	Plant-Mediated	Microbial-Mediated (Bacteria and Fungi)	Algae-Mediated	Biopolymer/Natural Molecule-Mediated	References
**Atom economy (AE)**	Moderate–high (extracts act as both reducing and capping agents, minimizing extra reagents)	Moderate (nutrient media adds to inputs, lowering AE)	High (algal metabolites directly reduce/cap NPs)	Moderate–high (efficient use of natural molecules, but polymer residues may persist)	[71,72,73]
**E-Factor (Waste/product ratio)**	Medium (biomass waste and extract residues)	High (culture media, biomass disposal)	Low–medium (minimal waste, scalable with photobioreactors)	Medium–high (non-biodegradable polymer residues possible)	[74]
**PMI (process mass intensity)**	Moderate (large extract volumes needed)	High (nutrient-rich media per product yield)	Low (aqueous systems, minimal reagents)	Medium (depends on polymer/natural molecule load)	[69]
**Energy consumption**	Moderate (room-temp synthesis, but extraction may require heating/solvents)	High (long culture times, controlled growth conditions)	Low–moderate (uses sunlight/photosynthesis, but harvesting biomass may be energy-intensive)	Low–moderate (simple mixing, but some polymer processing may require energy)	[30]
**Toxicity/hazard profile**	Low (plant metabolites are generally safe and biodegradable)	Low–Moderate (safe metabolites, but endotoxins/fungal toxins possible)	Very low (nontoxic algal biomolecules)	Moderate (biopolymers safe, but chemical crosslinkers or modified natural molecules may introduce toxicity)	[75]
**Overall greenness**	Good (widely scalable, eco-friendly but extract waste handling needed)	Moderate (effective but resource-intensive)	Excellent (renewable, low waste, safe byproducts)	Good (sustainable, but residues and chemical modifications reduce greenness)	[30]

**Table 2:** Summary of ROS-induced cellular damage by green-synthesized NPs.

Effect	Description	References
Lipid peroxidation	Damages microbial membrane integrity by attacking phospholipid bilayers; causes membrane leakage	[106]
Protein oxidation	Inactivates key enzymes and structural proteins; disrupts metabolism and cell signaling	[107]
DNA/RNA damage	Causes base modifications, strand breaks, and replication failure, leading to mutations or cell death	[108]
ROS-induced apoptosis-like death	Triggers programmed like cell death, especially in fungi and protozoa; involves oxidative stress pathways	[109]

**Table 3:** Influence of capping agents on ROS generation and metal ion release in green-synthesized nanomaterials.

Capping Agent	Effect on ROS Generation	Effect on Metal Ion Release	Representative Quantitative Examples	Implications for Water Disinfection and Safety	References
Polyphenols (e.g., flavonoids, tannins, phenolic acids)	Can enhance interfacial ROS via redox cycling at the NPs surface; act as bulk antioxidants, reducing detectable ROS in solution.	May stabilize NPs surface (slower release) or promote dissolution via soluble polyphenolmetal complexes; outcome depends on ligand chemistry and environment.	AgNPs (polyphenol-capped): ROS ≈ 0.25–0.35 µmol H_2_O_2_/min/mg NPs; MIC ≈ 350–500 mg·L^−1^ *coli*, *S. aureus*). ZnO phenolic NPs: ~15–20% higher ROS vs. uncapped ZnO.	Strong contact-mediated antimicrobial action; but variable ion release may affect long-term safety in aquatic environments.	[121]
Proteins (e.g., albumin, microbial proteins, enzymes)	Generally, reduce both interfacial and bulk ROS due to steric shielding and amino acid scavenging.	Strong binding (cysteine, histidine) typically suppresses acute ion release; release may increase if corona degrades in natural waters.	AgNP@BSA: Lower ROS vs. ligand-capped AgNPs; MIC ≈ 15 mg·L^−1^ (*E. coli*) vs. 63 mg·L^−1^ (uncapped). ICP-MS: protein-capped AgNPs released ~40–50% less Ag^+^ after 24 h vs. uncapped.	Offers lower immediate ROS/ion toxicity and enhanced colloidal stability, but corona degradation can lead to delayed ion release.	[122]
Polysaccharides (e.g., starch, chitosan, alginate)	Typically, moderate ROS generation; chitosan can impart intrinsic antimicrobial effects (membrane disruption).	Often reduce ion release by forming dense coatings, though porous polysaccharide shells (e.g., alginate) may allow gradual release.	Starch-capped AgNPs: MIC ≈ 25–30 mg·L^−1^ (*E. coli*). Chitosan–ZnO NPs: ~30% lower Zn^2+^ release vs. bare ZnO after 48 h.	Provide sustained ion release and improve biocompatibility; chitosan adds dual-function antimicrobial action.	[117]
Lipids/Terpenoids (e.g., plant essential oils, saponins)	Can act as ROS quenchers (antioxidant terpenoids) or enhance ROS under light via photosensitization.	Lipid layers typically slow ion release, but terpene-metal complexes may promote dissolution under certain pH conditions.	AgNPs capped with terpenoid-rich extract: MIC ≈ 20–40 mg·L^−1^ (*S. aureus*). Essential oil–capped NPs showed ~25% lower ROS vs. polyphenol-capped analogs in DCFH assays.	Add synergistic antimicrobial properties (membrane disruption), but ROS suppression may limit oxidative disinfection pathways.	[123]

**Table 4:** Summary of typical protocols and evaluation metrics for GSNMs.

Category	Reported Practices
**Synthesis**	Biological sources include plants, microbes, algae, and natural polymers. Metal precursors commonly include AgNO_3_, zinc acetate, and titanium salts. Reactions are generally performed under mild conditions, with pH, temperature, and precursor concentration influencing nanoparticle size and shape. Visible color change (e.g., yellow to brown for AgNPs) is often noted as a first indicator of nanoparticle formation.
**Characterization**	Morphology and size are evaluated by electron microscopy techniques (e.g., TEM, SEM). Crystalline structure is confirmed using XRD. Surface functional groups involved in stabilization are identified by FTIR. Colloidal stability is assessed through zeta potential measurements.
**Antimicrobial Testing**	Agar well/disk diffusion assays are used to determine inhibition zones (mm). MIC and MBC values are reported to quantify activity (commonly expressed in mg·L^−1^ or µg·mL^−1^). Microdilution assays and CFU counts are applied for bacterial viability assessment. Some studies also evaluate ROS generation and cell membrane damage as possible mechanisms.

**Table 5:** Comparative performance of green-synthesized nanomaterials in different water matrices.

Water Type	Key Characteristics	Waste Removal Efficiency	References
**River/surface water**	Moderate turbidity (≤50 NTU), NOM present (humic/fulvic acids)	>90% bacterial inactivation by green AgNPs up to 50 NTU; efficacy decreases with high NOM fouling	[160,161]
**Groundwater**	Low turbidity, low NOM, stable pH	Very high removal at MIC doses; stable efficacy with AgNPs, ZnO, TiO_2_	[163,164]
**Wastewater**	High NOM, high turbidity, organic/inorganic load	Reduced direct NP efficacy; requires pre-treatment or photocatalytic activation for >80% removal	[100,165]

**Table 6:** Comparison of CSNPs and GSNPs in terms of their long-term environmental fate, cytotoxicity, ROS production, and antibacterial activity.

Nanoparticle Type	Synthesis Route	Antimicrobial Efficacy	Cytotoxicity in Mammalian Cells	ROS Generation and Modulation	Environmental Fate and Long-Term Risk	References
**ZnO NPs**	GSNP (e.g., *Musa acuminata* extract)	Strong antibacterial, enhanced under UV due to photocatalysis	Moderate, dose and time dependent	Elevated ROS but moderated by bioorganic capping	Reduced ion release (Zn^2+^); lower sediment toxicity	[180]
**ZnO NPs**	CSNP (precipitation/sol–gel)	Comparable antimicrobial activity	High; apoptosis at lower doses	High ROS without moderation	Significant Zn^2+^ leaching; higher long-term ecotoxicity	[181]
**Ag NPs**	GSNP (e.g., *Euphorbia retusa*, *Beta vulgaris*)	Potent antimicrobial, effective vs. MDR strains	Moderate; apoptosis at higher concentrations	ROS partly mitigated by plant polyphenols	Lower Ag^+^ release; reduced bioaccumulation risk	[182]
**Ag NPs**	CSNP (NaBH_4_, citrate reduction)	Very high antimicrobial activity	High; mitochondrial dysfunction and DNA damage	Excess ROS; no antioxidant regulation	High Ag^+^ release; higher bioaccumulation risk	[120]
**CuO NPs**	GSNP (microbial/algal synthesis)	Effective antibacterial, Fenton-like ROS production	Lower cytotoxicity than chemical CuO	ROS moderated by organic capping	Partial dissolution; sediment accumulation potential	[11]
**CuO NPs**	CSNP (Thermal decomposition)	High antimicrobial activity	Pronounced cytotoxicity; oxidative stress	Persistent high ROS from surface defects	High dissolution rate; elevated aquatic toxicity	[114]
**Fe_3_O_4_ NPs**	GSNP (microbial/algal synthesis)	Good antibacterial; magnetically retrievable	Biocompatible at moderate doses	ROS less toxic due to capping	Partially retrievable; sediment accumulation possible	[95]
**Fe_3_O_4_ NPs**	CSNP (co-precipitation)	Effective antibacterial	Higher cytotoxicity; stress from surface residues	ROS enhanced by surface defects	Sediment persistence; stronger ecotoxic impact	[183]

## Data Availability

No new data were created or analyzed in this study. Data sharing is not applicable to this article.
